# Prognostic Biomarkers in Uveal Melanoma: The Status Quo, Recent Advances and Future Directions

**DOI:** 10.3390/cancers14010096

**Published:** 2021-12-25

**Authors:** Nuno Jorge Lamas, Arnaud Martel, Sacha Nahon-Estève, Samantha Goffinet, Adam Macocco, Corine Bertolotto, Sandra Lassalle, Paul Hofman

**Affiliations:** 1 Laboratory of Clinical and Experimental Pathology, Université Côte d’Azur, Pasteur Hospital, Centre Hospitalier Universitaire de Nice, Biobank BB-0033-00025, 06000 Nice, France; goffinet.s@chu-nice.fr (S.G.); adam.macocco@hotmail.fr (A.M.); lassalle.s@chu-nice.fr (S.L.); 2 Anatomic Pathology Service, Pathology Department, Centro Hospitalar e Universitário do Porto, Largo Professor Abel Salazar, 4099-001 Porto, Portugal; 3 Life and Health Sciences Research Institute (ICVS), School of Medicine, University of Minho, Campus de Gualtar, 4710-057 Braga, Portugal; 4 ICVS/3B’s-PT Government Associate Laboratory, University of Minho, 4710-057 Braga, Portugal; 5 IRCAN Team 4, Inserm U1081/CNRS 7284, Centre de Lutte Contre le Cancer Antoine Lacassagne, 06000 Nice, France; martel.a@chu-nice.fr (A.M.); nahon-esteve.s@chu-nice.fr (S.N.-E.); 6 FHU OncoAge, Centre Hospitalier Universitaire de Nice, 06000 Nice, France; 7 Department of Ophthalmology, Pasteur Hospital, Centre Hospitalier Universitaire de Nice, 06000 Nice, France; 8 Department of Biology and Pathologies of Melanocytes, Team1, Equipe Labellisée Ligue 2020 and Equipe Labellisée ARC 2019, Centre Méditerranéen de Médecine Moléculaire, INSERM, 06200 Nice, France; corine.bertolotto@unice.fr

**Keywords:** uveal melanoma, prognostic factors, biomarkers, metastases, survival, molecular pathology

## Abstract

**Simple Summary:**

Although rare, uveal melanoma (UM) is the most common cancer that develops inside adult eyes. The prognosis is poor, since 50% of patients will develop lethal metastases in the first decade, especially to the liver. Once metastases are detected, life expectancy is limited, given that the available treatments are mostly unsuccessful. Thus, there is a need to find methods that can accurately predict UM prognosis and also effective therapeutic strategies to treat this cancer. In this manuscript, we initially compile the current knowledge on epidemiological, clinical, pathological and molecular features of UM. Then, we cover the most relevant prognostic factors currently used for the evaluation and follow-up of UM patients. Afterwards, we highlight emerging molecular markers in UM published over the last three years. Finally, we discuss the problems preventing meaningful advances in the treatment and prognostication of UM patients, as well as forecast new roadblocks and paths of UM-related research.

**Abstract:**

Uveal melanoma (UM) is the most common malignant intraocular tumour in the adult population. It is a rare cancer with an incidence of nearly five cases per million inhabitants per year, which develops from the uncontrolled proliferation of melanocytes in the choroid (≈90%), ciliary body (≈6%) or iris (≈4%). Patients initially present either with symptoms like blurred vision or photopsia, or without symptoms, with the tumour being detected in routine eye exams. Over the course of the disease, metastases, which are initially dormant, develop in nearly 50% of patients, preferentially in the liver. Despite decades of intensive research, the only approach proven to mildly control disease spread are early treatments directed to ablate liver metastases, such as surgical excision or chemoembolization. However, most patients have a limited life expectancy once metastases are detected, since there are limited therapeutic approaches for the metastatic disease, including immunotherapy, which unlike in cutaneous melanoma, has been mostly ineffective for UM patients. Therefore, in order to offer the best care possible to these patients, there is an urgent need to find robust models that can accurately predict the prognosis of UM, as well as therapeutic strategies that effectively block and/or limit the spread of the metastatic disease. Here, we initially summarized the current knowledge about UM by compiling the most relevant epidemiological, clinical, pathological and molecular data. Then, we revisited the most important prognostic factors currently used for the evaluation and follow-up of primary UM cases. Afterwards, we addressed emerging prognostic biomarkers in UM, by comprehensively reviewing gene signatures, immunohistochemistry-based markers and proteomic markers resulting from research studies conducted over the past three years. Finally, we discussed the current hurdles in the field and anticipated the future challenges and novel avenues of research in UM.

## 1. Introduction

Uveal melanoma (UM) is the most common primary malignant neoplasia afflicting the eyes of adults [1,2]. Metastases develop in approximately 50% of patients, who then have a shortened life expectancy [1,2,3]. UM metastasis can develop up to 30 years after the initial diagnosis and treatment [4], and once metastases are detected, the median survival time for UM patients is approximately 12 months, especially because therapeutic options for advanced disease are limited and mostly ineffective [5,6,7]. The identification of robust clinical and molecular biomarkers that can accurately predict the prognosis of patients, namely, the possibility of metastases development, is therefore of extreme relevance and an ongoing challenge in the field [8]. The discovery of robust prognostic biomarkers and/or models has the prospect to positively impact in a personalized UM patient approach, with patient-targeted surveillance and therapeutic strategies [9]. This is particularly pertinent since that there are diverse guidelines for the medical follow-up of UM patients and a definition of which tests are the most effective in detecting early disease relapse is lacking [for example, should patients be followed using only liver ultrasound and/or magnetic resonance imaging (MRI)? Should the MRI be performed with or without contrast medium?] [9,10]. On one hand, the establishment of UM patients with high-risk of disease relapse would lead to a more close monitoring of those patients [11]. On the other hand, the early detection of disease relapse, such as liver metastases, could enable the surgical removal or chemoembolization of those lesions in a premature state, which seems to be the most efficacious strategy currently available to deal with metastatic disease and to extend the life of UM patients with advanced disease [7,10,12]. In addition, liver lobe resection, systemic chemotherapy, radiofrequency ablation or isolated liver perfusion constitute alternative therapeutic approaches for UM metastatic to the liver, however, are essentially unsuccessful at achieving a final cure for the patient [6,12,13]. It is possible that patients who are eligible for metastases resection have a lower burden of disease and a potentially more favourable tumour biology compared with UM patients non-eligible for metastatic ablation [14].

In the present review, we first appraise the main clinical, epidemiological and pathological features of UM. Next, we review the aspects of UM genetics which are at the core of neoplastic transformation and summarize the most relevant knowledge on the currently used prognostic markers in UM, including the updated views on the molecular classification of UM. Afterwards, we review the gene signatures and novel immunohistochemistry-based biomarkers with prognostic relevance in UM published over the past three years. Finally, we discuss the current hurdles in the field, imminent challenges and the promising future research avenues towards a successful and optimized treatment of patients afflicted by this aggressive disease that significantly reduces the quality of life and average life expectancy of patients.

## 2. Uveal Melanoma: Relevant Epidemiological, Clinical and Pathological Features

**Figure 1:**
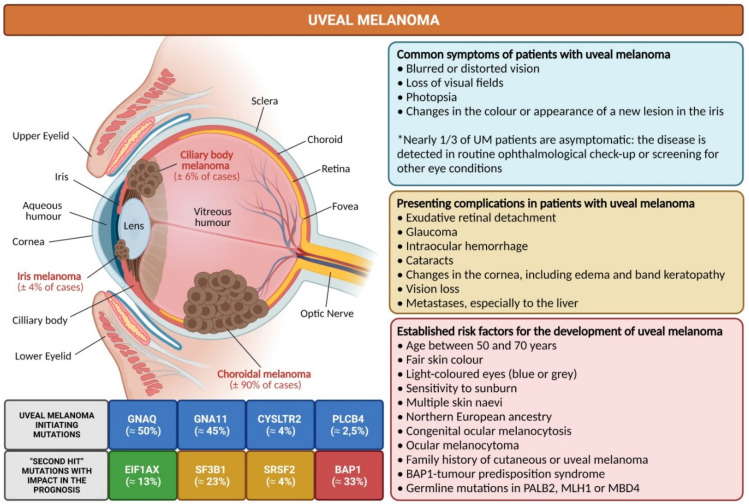
Overview of key facts in uveal melanoma, the most common intraocular primary malignant tumour in adults. Different risk factors are associated with the development of uveal melanoma. The choroid is the most frequent intraocular site of uveal melanoma development, which is detected in routine ophthalmological exams in asymptomatic patients. However, the majority of uveal melanoma patients present with symptoms, such as blurred vision or photopsia. Metastases, especially to the liver, occur in nearly 50% of patients during the first 10 years after diagnosis, but constitute a presenting symptom in only a small fraction of patients (<2%). In the carcinogenic process of uveal melanoma, several tumour-initiating and tumour-promoting mutations have already been identified and characterized. Uveal melanoma patients with mutations in BAP1 (highlighted in red) have been demonstrated to have the worst outcome, while patients with EIF1AX (highlighted in green) have a better prognosis and patients with SF3B1/SRSF2 (highlighted in orange) have an intermediate prognosis. Diagram generated in line with previous literature [1,2,3,9,22,25,26,27,36,37,38,39,40] (Diagram created with BioRender.com, accessed on 15 December 2021).

The first known description of the complete natural history of UM dates back to the beginning of the 19th century, when two Scotland-based surgeons, Allan Burns and James Wardrop, described and detailed the clinical history of a 41-year-old woman living in Glasgow, who developed an intraocular lesion that rendered her quickly blind and which became extremely painful and with extrascleral extension only after 4 months [15]. Even though she was enucleated, the patient later evolved with hepatic and abdominal metastases and died in less than a year after the initial medical visit [15]. UM develops from the uncontrolled proliferation of melanocytes in the uveal tract, which comprises the pigmented tissues in both the anterior (iris) and the posterior (choroid and ciliary body) segments of the eye (Figure 1). The disease is usually unilateral and the majority of UM cases have their epicenter in the choroid (≈90%), while nearly 6% of them are restricted to the ciliary body and 4% to the iris (Figure 1) [1,3,16]. The annual incidence in Europe and USA is ≈5 per million population, but worldwide it can range from <1 to >9 per million population per year [17,18,19,20]. Most patients with UM are diagnosed between 50 and 70 years old [18,21]. The symptoms that most commonly prompt UM patients for a medical visit are blurred or distorted vision, loss of visual fields, photopsia and changes in the colour or appearance of a new lesion in the iris (Figure 1) [9,22]. However, in nearly one-third of the UM cases, the patients are asymptomatic and the disease is only detected due to routine ophthalmological check-up or screening for other eye conditions, such as diabetic retinopathy (Figure 1) [9,22]. The most common presenting complications encountered in patients with UM are exudative retinal detachment, glaucoma, cataracts, intraocular hemorrhage, vision loss, changes in the cornea including edema and band keratopathy [21,22,23]. Less than 2% of UM patients have long-distance metastases already at presentation (Figure 1) [24].

Over the past years, numerous risk factors have been described as being associated with the development of UM (Figure 1), of which the most established are an age between 50 and 70 years, a fair skin colour, light-coloured eyes (blue or grey), multiple skin naevi, sensitivity to sunburn, northern European ancestry, congenital ocular melanocytosis, ocular melanocytoma, family history of cutaneous melanoma or UM, BAP1 (BRCA1-associated protein 1)-tumour predisposition syndrome and also germline mutations in MBD4 (methyl-CpG-binding domain protein 4), MLH1 (mutL homolog 1) and PALB2 (partner and localizer of BRCA2) [1,9,25,26,27]. Interestingly, in UM there is no evidence of gene signatures indicative of tumours induced by ultraviolet (UV) irradiation, with the only exception being iris melanoma and some residual cases of posterior melanomas [28,29,30].

Once the diagnosis of UM is made, the treatment will aim to treat the tumour, preserve the eye up to its best functional state possible and conserve the vision [9]. Therapeutic modalities include phototherapy (no longer recommended), different forms of radiotherapy (^106^ruthenium brachytherapy or ^125^iodine brachytherapy, proton beam therapy or stereotactic radiosurgery) and local resection after radiotherapy for selected lesions [9,31,32,33,34,35]. Besides this, enucleation is the most adequate option for large-sized UMs and cases with advanced local disease, since it allows an enhanced local control with improved quality of life for the UM patient [9,31,32,33,34,35]. Interestingly, previous studies demonstrated superimposable mortality rates when comparing proton beam irradiation versus enucleation for patients with large choroidal melanomas [33]. Local tumour control is achieved in more than 95% of cases, even for large-size tumours [1,9]. Despite the successful local disease control, metastases will develop in nearly 50% of patients during the first 10 years (Table 1) [3,16].

Death related with metastases onset is more likely in the first 10 years after UM diagnosis, being scarcely observed beyond 20 years after the initial treatment [46,47,48]. Some studies demonstrate that metastization might occur early in the disease process and that micrometastases can remain in a quiescent state for several years, without the possibility of efficiently detecting them, similarly to other malignant neoplasias [49,50,51]. The liver (≈85%) is the preferred site for metastases development in UM, sometimes constituting the initial presentation of the disease [41,52] (Table 1). Other locations of UM metastasis include the lung (≈29%) and bone (≈16%), among others [24,41] (Table 1). In nearly one-third of the metastatic cases, there is involvement of multiple sites by UM metastases [24,41]. Despite the advances in the understanding of UM biology and the advent of new therapeutic modalities, the 5-year survival rate for UM (≈80%) has remained stable over the past five decades [46,47,48].

## 3. The Main Genetic Features of Uveal Melanoma

The genetic studies conducted over the past couple of decades allowed the identification of significant mutations in almost a dozen of genes which are relevant for UM development (Figure 1) [36,53]. In contrast to other tumours, including cutaneous melanoma [54] or lung adenocarcinoma [55], the tumour mutational burden (TMB; defined as the number of non-inherited mutations per million of bases of investigated genomic sequence) of UM is normally low [28,36,40]. Indeed, The Cancer Genome Atlas (TCGA) project (http://cancergenome.nih.gov/, accessed on 15 December 2021) showed that TMB for UM was 1,1 per Mb, whereas for cutaneous melanoma it was 18 per Mb [40,56]. In UM, the genes whose studies demonstrated the presence of mutations which are relevant for UM development can fundamentally be grouped into genes with tumour-initiating mutations and genes harbouring mutations with relevant impact in the prognosis of patients (Figure 1) [36,53,57]. Indeed, more than 90% of the patients have activating mutations in GNAQ (Guanine nucleotide-binding protein G(q) subunit alpha, ≈50%) and GNA11 (Guanine nucleotide-binding protein subunit alpha-11, ≈45%) (Figure 1) [36,53]. These mutations are normally mutually exclusive, which means that if a patient carries a mutation in GNAQ, they normally do not harbour a mutation in GNA11 and vice versa [36,58]. GNAQ and GNA11 encode proteins that are both involved in the Gα11/Q pathway, which regulates a myriad of cellular processes, including cell proliferation and growth [58,59,60]. In a restrict number of UM patients, there are tumour initiating mutations in CYSLTR2 (cysteinyl leukotriene receptor 2), which also encodes a G-protein coupled receptor (Figure 1) [61,62], or PLCB4 (1-Phosphatidylinositol-4,5-bisphosphate phosphodiesterase beta-4), which encodes a protein downstream in the GNAQ signalling cascade (Figure 1) [63]. Together, all these tumour-initiating mutations suggest that a dysregulated G-protein signalling is at the core of the carcinogenic process in UM development [58]. However, these mutations do not differentially impact on the prognosis of UM [64]. Recent research proposed that the pathway based on the axis GNAQ/11–PLCβ–PKC–MAPK could be a preferential target in the treatment of tumours with underlying Gαq pathway mutations, such as most of the UM cases [65]. Interestingly, UM normally arise de novo, but they can also develop from choroidal nevi, which frequently contain mutations in GNAQ, GNA11, CYSLTR2 and PLCB4 [62,66].

The process of malignant transformation in UM critically depends on “second hit” mutations, which in addition will also considerably impact on the prognosis of patients (Figure 1) [36,57,67]. Indeed, several studies suggest that metastization in UM is an early event and the ability to develop metastases with clinical impact is directly linked with the “second hit” genetic alterations of the primary tumour [36,57,67]. The most important mutated genes included in this group are BAP1, EIF1AX (eukaryotic translation initiation factor 1A, X-chromosomal), SF3B1 (splicing factor 3B subunit 1) and SRSF2 (serine and arginine rich splicing factor 2) (Figure 1) [36,67]. Nearly 10 years ago, in a breakthrough study, researchers identified in 26 of 31 (84%) metastasizing UM cases inactivating somatic mutations in the gene encoding BAP1 [48], which is located on chromosome 3p and is a deubiquitylase that participates in molecular complexes that are pivotal to the regulation of cell cycle, cellular differentiation, cell death and DNA damage response (DDR), among other key cellular pathways [49]. This study implicated that loss of BAP1 is a major event in the development of UM metastases [48]. Subsequent research demonstrated germline BAP1 mutations with familial clustering in different neoplasias, leading to the discovery of a new cancer syndrome, termed BAP1-tumour predisposition syndrome, which leads to the development of benign and malignant melanocytic skin tumours, malignant mesothelioma, UM and renal cell carcinomas, among other neoplasias [41,49,50,51,52]. BAP1 mutations are identified in nearly one-third of UM cases and loss of BAP1 or partial deletion of chromosome 3 including the BAP1 locus is a stronger predictor of higher risk of metastases and poor survival for UM patients [36,67,68].

The EIF1AX gene is located on chromosome X [53,69] and encodes a protein that interacts with mRNA, being involved in translation initiation, by a combination of recognition of target mRNA and also of ribosome stabilization, preparing mRNA for translation [53,69]. Mutations in EIF1AX, which occur in nearly 13% of UM cases (Figure 1), appear to be mutually exclusive to SF3B1 in UM and lead to altered protein translation processes [36,69]. Patients who harbour theses mutations have a decreased risk of metastases development and, therefore, a considerable better prognosis comparatively to UM patients with BAP1 loss (Figure 1) [36,67,70].

SF3B1 gene is located in chromosome 2 and is responsible for encoding the subunit 1 of the splicing factor 3b protein complex, a large molecular apparatus which is involved in the processing of precursor mRNA (spliceosome) [53,71]. It guarantees that correct splicing occurs through retaining pre-mRNA to define the site for splicing [53,70]. SF3B1 mutations, found in nearly 20% of UM cases (Figure 1), can therefore lead to alternative splicing events for a myriad of genes [69,70]. On the other hand, the SRFS2 gene is located in chromosome 17 and is a member of the serine/arginine (SR)-rich family of pre-mRNA splicing factors, which constitute part of the spliceosome [53,72]. Mutations in SRSF2 are found in up to 4% of UM cases (Figure 1) [36]. Similarly to SF3B1, mutations in SRFS2 lead to alternatively spliced transcripts [70,72]; however, the details of the impact of SF3B1/SRFS2 mutations in UM remain to be entirely understood [53,72]. Patients who harbour SF3B1/SRFS2 mutations have an increased risk of late-onset metastasis and, thus, have an intermediate prognosis comparatively to UM patients with EIF1AX mutations (low-risk) and BAP1 loss (high-risk) (Figure 1) [36,67,70,71].

## 4. Current Well-Established Prognostic Biomarkers in Uveal Melanoma

The establishment of an accurate prognosis for patients with UM is pivotal [73,74]. In this regard, the prognostic class of a given patient could impact on the specific protocol for surveillance of metastases development [8,11]. In addition, the stratification of patients based on their risk of metastases development or death could be a vital tool to select candidates to be included in clinical trials aiming to test promising adjuvant therapies [8,11,75]. In addition, patients might wish to know their accurate prognosis, which could be important in end-of-life planning, especially in a disease with no currently approved standard therapy for metastatic disease, which normally is associated with an extremely dismal prognosis [5,7,75,76].

Numerous robust prognostic factors for UM were established over the past years and are currently taken into account when evaluating primary UM cases (Table 2). Age could play an important prognostic role in UM, since adults older than 60 years at the time of diagnosis have an enhanced risk of metastases development compared to young and middle-aged adult patients [77,78]. Interestingly, some studies also suggest that gender could influence the prognosis of UM patients, documenting a worse prognosis for males, who have increased rate of metastases development and a decreased survival in the first decade after UM diagnosis [17,79].

**Figure 2:**
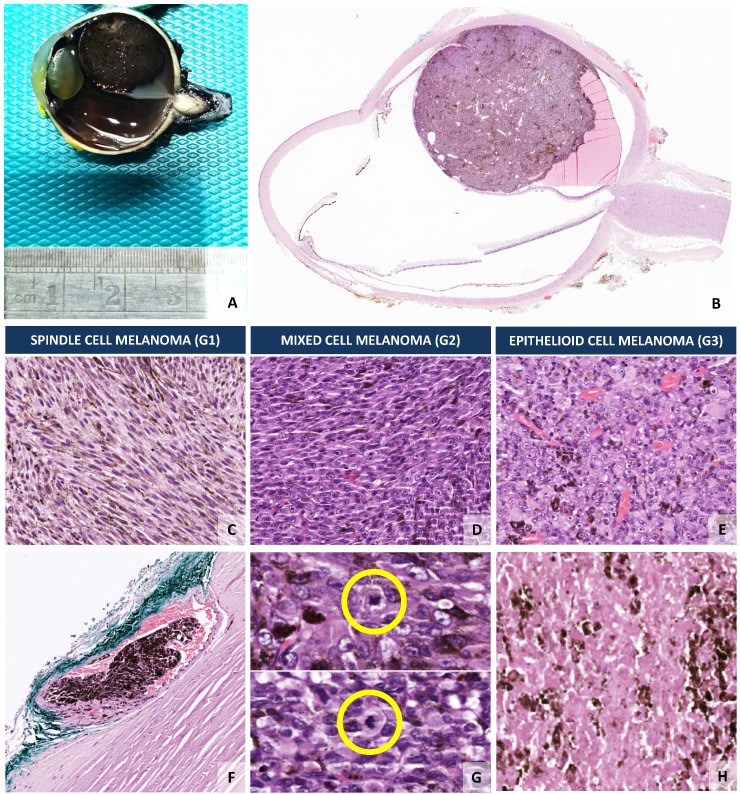
Uveal melanoma is a primary malignant tumour of the eye with a potential dismal prognosis, since nearly 50% of the patients die because of metastases, preferentially to the liver, which are not curable due to the absence of meaningful therapeutic strategies. The morphological features of uveal melanoma are instrumental to predict the prognosis of patients. (**A**) Eye specimen containing a pigmented round tumour located in the choroid (posterior segment of the eye), the most frequent anatomic location of uveal melanomas. (**B**) Whole-slide representative microscopic view of the large-sized choroidal melanoma with evidence of associated exudative retinal detachment (H&E, 2× magnification). (**C**) Uveal melanomas composed by more than 90% of spindle cells are called spindle cell melanomas (G1; H&E, 400× magnification). (**D**) Uveal melanomas containing more than 10% of a spindle cell component and less than 90% of an epithelioid component are termed mixed cell melanomas (G2; H&E, 400× magnification). (**E**) Epithelioid cell melanomas (G3), which are associated with a worse patient prognosis, are composed by more than 90% of epithelioid malignant cells (H&E, 400× magnification). (**F**) Uveal melanoma disseminates systemically through a preferential haematogenous pathway. The presence of images of vascular invasion is correlated with a worse prognosis for patients (H&E, 200× magnification). (**G**) The presence of an increased number of mitosis (yellow circle) also hints a worse outcome for uveal melanoma patients (H&E, 200× magnification). (**H**) The presence of necrosis in non-treated uveal melanoma cases is an additional marker of bad prognosis for patients (H&E, 200× magnification). A summary of all currently well-established markers of bad prognosis in uveal melanoma is presented in Table 2.

The location of the UM within the eye also has important prognostic implications [16,80]. While UM centred in the ciliary body or involving the ciliary body have the worst mortality rates within 5 years after diagnosis, iris melanoma has the best prognosis, with some studies pointing towards a 10-year mortality below 10% [9,81]. Choroidal melanoma, which is the most frequent type of UM (Figure 2), has an intermediate prognosis between ciliary body and iris melanoma [81]. The best prognosis for iris melanoma can be linked with the fact that the iris is easily visualized and most lesions are detected at an early stage [9,81,82]. Conversely, the ciliary body has a localization that is challenging for clinical examination, which can only be accomplished by slit-lamp examination, ophthalmoscopy, gonioscopy, or transillumination, so that lesions tend to grow larger before they can be detected [9,82]. In addition, the possibility of invasion of the Schlemm’s canal allows an easier and faster route for systemic dissemination of the UM [83,84].

The tumour size is among the most robust established prognostic factors in the UM medical literature [8,80]. The largest basal diameter (LBD) and tumour thickness (TT), which are more accurately measured by ultrasonography and fundus photography, help to stratify the size of UM cases in small (T1), medium (T2), large (T3) and very large (T4) tumours, which considerably differ in their survival prognosis [73,82,85]. Some early studies showed that patients with tumours with a LBD above 15 mm have a significantly shorter survival comparatively to patients with tumours smaller than 15 mm [86]. In the international study for validation of the 7th edition of the American Joint Committee on Cancer (AJCC) classification for UM, involving more than 3000 patients, the authors performed Kaplan–Meier metastases-free estimates (5, 10 years), obtaining the following results: T1 (97%, 94%), T2 (85%, 80%), T3 (77%, 68%) and T4 (61%, 5-year only) [85]. In addition, in a previous study involving a large cohort of 8033 UM patients, the authors demonstrated a significant 5% increase in the risk of metastases at 10 years per each millimetre of increased thickness of the UM [16]. Therefore, increased UM size indicates a worse patient prognosis [16,85].

**Table 2:** Currently well-established prognostic factors in primary uveal melanoma. All the factors highlighted below are associated with a worse prognosis for patients diagnosed with UM.

Factors Associated with a Worse Prognosis for Uveal Melanoma Patients
Higher age at diagnosis [77,78]
Male gender [17,79]
Ciliary body location and involvement [9,81,82]
Increased tumour size [Largest basal diameter (LBD) and tumour thickness (TT)] [73,82,85]
Epithelioid cell morphology [8,80,87]
Vascular invasion [83,88]
Extraocular spread [84,89]
Increased mitotic count [87,90,91]
Increased microvessel density [92,93]
Presence of tumour-infiltrating lymphocytes (TILs) [94,95,96]
Presence of tumour-infiltrating macrophages (TIMs) [94,95,96,97]
Presence of necrosis (in non-treated UM) [81]
Higher T stage (AJCC, TNM staging) [73,98]
Presence of uveal melanoma metastases [7,8,98]
Loss of nuclear BAP1 expression/BAP1 mutation [99,100,101,102]
PRAME expression [103,104,105,106]
Chromosomal abnormalities, especially M3, 8q gain, 6q loss and 1p loss [3,36,39,40,73,107,108,109]
Gene Expression Profiling (GEP) Class 2 [110,111,112,113]

The morphology of the UM cells also has important prognostic implications for ciliary body and choroidal melanoma (Figure 2), whereas in iris melanoma this factor has no prognostic implications [8,80]. Patients with spindle cell UM, which contains more than 90% of spindle cells (G1) have the best survival; while epithelioid cell melanomas, which comprises more than 90% epithelioid cells (G3), have the worst prognosis (Figure 2) [8,114]. The melanomas which contain less than 90% of spindle cells and more than 10% of epithelioid cells (G2), termed mixed cell melanomas, have an intermediate prognosis (Figure 2) [8,114]. Interestingly, some authors demonstrated that the presence of any percentage of an epithelioid component is *per se* an indicator of a worse outcome [87].

UM preferentially escapes from the eye microenvironment through a haematogenous pathway [115]. The process seems to involve transendothelial migration using complex mechanisms of ameboid blebbing and mesenchymal lamellipodial protrusion, which remain to be fully understood [115]. Dissemination through the lymphatic system only occurs if there is extraocular extension with invasion of the conjunctival lymphatics [116,117]. Therefore, UM spread is intimately linked with the presence of tumour cells in blood vessels (Figure 2) [83], so that the presence of images of vascular invasion in UM (Figure 2), either inside or outside the tumoural area, is associated with a worse prognosis for UM patients and is correlated with other prognostic factors, such as LBD or epithelioid cell phenotype [88]. Additionally, the presence of angiotropism, which is defined as the presence of neoplastic cells disposed along the abluminal surface of vascular structures without intravasation, has also been demonstrated to be a prognostic factor for metastasis and UM-related death [83]. In line with this, microvessel density (MVD), a surrogate marker of angiogenesis which can be easily assessed using antibodies against CD34, became established as a significant prognostic factor in UM nearly two decades ago [92,93]. Higher MVD is independently associated with a poor prognosis and other markers of bad prognosis, such as epithelioid cell morphology and LBD [92,93], as well as with the UM genetic profile, namely, monosomy 3 [complete loss of one copy of chromosome 3 (M3)] and loss of BAP1 expression [118].

Extraocular spread of UM, irrespective of the type of extraocular spread route and dimension of the extraocular tumoural fragment, is a marker of worse prognosis in UM, being correlated with increased rate of metastases development and increased UM-related death [84]. Apparently, it is an indirect sign of enhanced tumour malignancy and for posterior tumours signals a more advanced disease state [84]. Extraocular spread is correlated with other important prognostic factors, such as UM size, tumour location, histologic type and cytogenetics [84,89]. For example, in UM cases with extraocular extension, a gain of chromosomal 8q is associated with increased risk of metastatic disease [89].

Mitotic counts constitute another robust and important classical prognostic factor in UM (Figure 2). In a straightforward manner, in haematoxylin and eosin (H&E) stained sections, mitotic counts are usually performed in 40 fields at high-power (40× objective), with or without the aid of immunohistochemistry (IHC) proliferation markers, such as Ki-67 or PHH3 (phospho-histone 3) [91]. Some pioneer studies demonstrated that mitotic counts were independently associated with metastatic risk and increased mitotic counts were correlated with a shorter survival [87,90].

The absence of a significant immune response against allografts placed within the ocular microenvironment led the pioneer transplantation immunologist Sir Peter Medawar to describe the eye as an immune privileged site nearly 70 years ago [119]. The eye has distinctive anatomical features, namely, a blood barrier analogous to the central nervous system (CNS) blood–brain barrier and lacks a direct lymphatic drainage [119,120]. Besides the anatomical features, novel distinctive immunological and biochemical mechanisms have emerged as plausible explanations for the immunologically unique and privileged microenvironment within the eye [119]. Therefore, unlike in other neoplasias, in UM patients the presence of tumour-infiltrating lymphocytes (TILs) and tumour-infiltrating macrophages (TIMs) is associated with a worse prognosis [94,95,96], with some authors suggesting it is a likely indirect signal of a disruption of the barrier between the eye microenvironment and the rest of the organism [121]. Studies in the early 1990s already demonstrated that patients with UM containing higher amounts of TILs had a worse outcome comparatively to patients with lower amounts of TILs [94,95]. In line with this, pioneer research conducted nearly 20 years ago also demonstrated that patients with UM containing higher amounts of TIMs had a shorter survival [96]. Ensuing studies showed that M2-type macrophages are the predominant macrophage population in UM, being more abundant in M3 UM cases comparatively to disomy 3 (D3) [97]. Interestingly, infiltration by M2-macrophages was linked with a shoddier prognosis for survival [97]. Our understanding of the interplay between the immune infiltrating cells and UM is still in its infancy, but as new knowledge emerges, a better understanding of the role and modulation of TILs and TIMs in UM might lead to innovative robust therapies for UM based on immunotherapy (please see Section 8) [122,123].

Similarly to other neoplasias, the presence of necrosis in non-treated tumours is also associated with an inferior prognosis in UM (Figure 2), with some studies demonstrating an association with other prognostic factors, namely, a correlation between a higher degree of necrosis and a larger tumour size, epithelioid morphology of UM cells or increased number of TIMs [81]. Therefore, the presence or absence of necrosis is a histomorphological feature that is normally assessed by the pathologist during the evaluation of UM cases (Figure 2).

UM is currently staged according to the 8th Edition of the TNM staging system of the American Joint Committee on Cancer (AJCC), which is still the gold standard system for prognostication in UM [73,98]. The TNM staging will help to define the follow-up strategies and the T category was demonstrated to be a robust predictor of UM metastatic disease with increased significant likelihood of metastases development at 5, 10 and 20 years for T1 (8%, 15%, 25%), T2 (14%, 25%, 40%), T3 (31%, 49%, 62%) and T4 (51%, 63%, 69%) stages [73,98]. In line with this, an analogous trend for significant increased risk of death with a higher T stage has also been demonstrated [73,98].

The development of a metastatic disease in UM is among the factors with the biggest impact in the definition of life expectancy of the UM patient [7,8]. Unless metastases are detected early and submitted to ablation therapy, the presence of metastases in UM is a marker of early death, given that effective therapeutic options for the metastatic UM disease are still limited [124,125]. In fact, recent studies demonstrate a median survival time of 17.5 months for M1a (largest diameter of the largest metastasis less than or equal to 3 cm), 9.6 months for M1b (largest diameter of the largest metastasis 3.1–8.0 cm) and 5 months for M1c (largest diameter of the largest metastasis greater than or equal to 8.1 cm) once metastatic UM disease is detected [98].

BAP1 status is one of the most relevant prognostic factors currently evaluated in patients with UM. Indeed, the determination of BAP1 status through IHC in primary UM has become routine in the prognostic evaluation, since it was shown to be highly correlated with gene mutation status [99,100,101,102]. Patients with loss of nuclear BAP1 staining were shown to have an 8-fold higher likelihood of developing metastases comparatively to patients with preserved nuclear BAP1 [100,101]. Therefore, BAP1 quickly became established as a robust independent survival predictor for UM patients, indicating the development of a likely aggressive metastatic phenotype [99,100,101]. Interestingly, a recent study also showed that BAP1 methylation at a single genomic locus is strongly correlated with BAP1 mutations, loss of BAP1 genomic copy and BAP1 protein levels [126]. Besides this, higher levels of BAP1 methylation significantly correlated with worse survival in UM patients [126]. A recent study also put in evidence that BAP1 mutations occur in the early steps of UM neoplastic development, before the tumour is even detected and with a timing that is likely to match the advent of the pioneer micrometastases [127].

Preferentially expressed antigen in melanoma (PRAME) was initially revealed by studies on skin melanoma as an antigen present in tumoural cells and recognized by T cells displaying cytotoxic activity [128]. In melanocytic lesions of the skin and conjunctiva, it is currently used as a helpful and robust adjunct marker to differentiate benign melanocytic lesions from melanoma [129,130]. Furthermore, the increased expression of PRAME is a marker of poor prognosis in different types of neoplasias, namely, breast cancer [131], head and neck squamous cell carcinoma (HNSCC) [132], neuroblastoma [133], osteosarcoma [134], among others. The prognostic significance of PRAME expression in UM emerged from a few studies conducted over the past 5 years, which showed that PRAME expression in UM is associated with an increased tumour volume, enhanced metastatic risk and global inferior prognosis [103,104,105,106].

**Figure 3:**
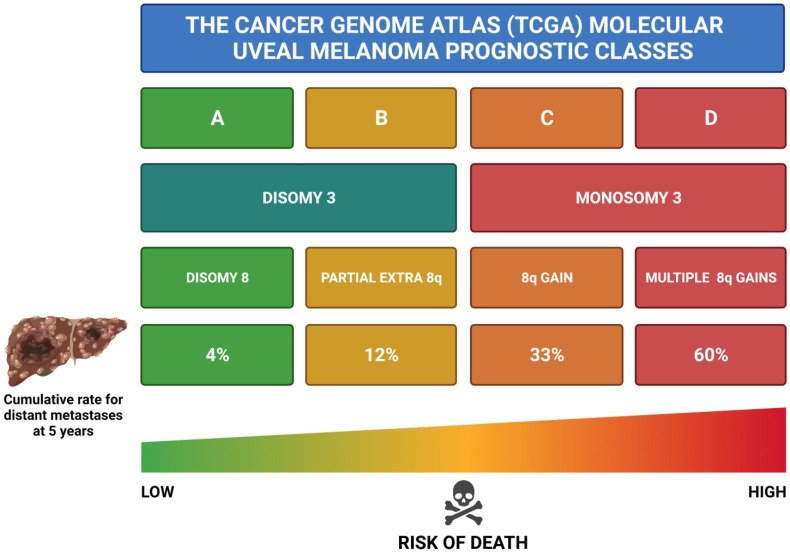
New molecular prognostic classification for uveal melanoma based on the data generated by the TCGA project involving primary uveal melanoma cases [40]. The new model comprises four main prognostic classes: class A [D3/D8], class B (D3/partial extra 8q), class C (M3/8q gain) and class D (M3/multiple 8q gains). The risk of metastases development increases progressively from class A to class D. Uveal melanoma patients in class D have the least favourable prognosis, with nearly all patients dying within the first decade after diagnosis. Diagram generated in line with previous literature [3,36,39,40,73] (Diagram created with BioRender.com, accessed on 15 December 2021).

Pioneer cytogenetic studies in the 1990s allowed the identification of important chromosomal abnormalities in UM which influence the prognosis of patients, especially involving chromosomes 1, 3, 6 and 8 [107,108,109]. The most important chromosomal abnormality is M3, which is strongly associated with a higher risk of metastases and, thus, a worse prognosis [40,73,135]. Regarding chromosome 1, total or partial loss of chromosome 1p is also a marker of poor prognosis, irrespective of the presence or absence of M3 [136,137]. As far as chromosome 6 is concerned, the gain of 6p is a robust indicator of good prognosis, since it has a reverse relationship with the metastatic risk [8,138]. On the contrary, the loss of chromosome 6q is correlated with a decreased life expectancy [8,138]. The loss of chromosome 8q is a rare event in UM, while gain of 8q is more common and linked with poor prognosis [8,73]. Interestingly, chromosome 8q gain frequently co-exists with M3 and these patients have the worst prognosis among all (Figure 3) [138,139]. In summary, M3, gain of 8q, loss of 1p and 6q loss are all associated with an inferior prognosis for UM patients [39,138,139]. A novel and more robust molecular prognostic classification of UM is being proposed, which has its backbone on chromosomal abnormalities (Figure 3) [36,40]. The new molecular classification is based on the data gathered from the TCGA project, in which a vast array of 80 UM patients had their primary tumour profiled through a comprehensive analysis involving different molecular methodologies (Figure 3) [39,40]. This new classification comprises four main prognostic classes: class A [D3/Disomy 8 (D8)], class B (D3/partial extra 8q), class C (M3/8q gain) and class D (M3/multiple 8q gains), with progressive increased risk of metastases development and, thus, increased risk of poor prognosis, from class A to class D (Figure 3) [3,36,39,40,73]. This new molecular classification has been demonstrated to be superior to the current gold standard AJCC TNM staging in predicting the risk of metastases and death (Figure 3) [3,36,73,139].

The risk of metastatic development for patients with UM can also be predicted using the Gene Expression Profiling (GEP; Castle Biosciences, Phoenix, AZ, USA) of the primary tumour, a commercially available test based on a 15-gene array conducted on a microfluidics quantitative polymerase chain reaction (PCR) platform, which allows accurate UM testing even from small needle biopsy samples [110,111]. A machine learning algorithm is then applied and stratifies UM patients into low metastatic risk (Class 1A), intermediate metastatic risk (Class 1B) and high metastatic risk (Class 2) [111,112,113]. This test has been validated by numerous studies [112,113] and, interestingly, a correlation between class 2 patients and loss of nuclear BAP staining/BAP1 mutation has been found [67,140]. The prognostic accuracy of GEP has been proven to be robust and superior to clinical features, histopathological analysis, TNM staging and evaluation of chromosomal abnormalities [112,141,142].

Worldwide, the usage of GEP and/or chromosomal analysis has been a heterogeneous scenario, with a few centres using both and several centres favouring the usage of one over the other. In centres with less abundance of resources, only the status of BAP1 is tested, through immunohistochemistry, as an indicator of BAP1 mutation and as a surrogate marker for M3. Finally, in a myriad of other centres none of these molecular markers are tested and, thus, UM patient prognostication still relies in classical histomorphological prognostic markers as described above (Table 2).

## 5. Gene Signatures as Novel Prognostic Biomarkers in Uveal Melanoma

Even though the current therapeutic options are effective in ablating local UM disease, invariably nearly half of the patients will develop metastases in the first decade after the initial diagnosis [9,10]. The ability to accurately predict the patients at high risk of metastases is, thus, of fundamental importance [8,143]. One of the main lines of investigation that the scientific community dedicated to the study of UM has followed is the development of genomic expression signatures of primary UM cases, aiming to find robust ones that can be used to construct reliable prognostic models which can be applied in the follow-up and treatment of patients diagnosed with UM (Table 3). Recently, these efforts were considerably accelerated following the public availability of the clinical and genomic datasets of the TCGA project (http://cancergenome.nih.gov/, accessed on 15 December 2021), in which a vast array of 80 UM patients had their primary tumour profiled [40]; and the datasets within the Gene Expression Omnibus (GEO) database (http://www.ncbi.nlm.nih.gov/geo/, accessed on 15 December 2021), such as GSE22138 (63 UM samples) [144], GSE27831 (29 UM samples) [145], GSE39717 (41 UM samples) [146] and GSE84976 (28 UM samples) [147]. Thus, several UM genomic databases are now available for usage by researchers worldwide, which has helped to significantly leverage genomic research in the UM field (Table 3).

In 2018, Wan et al. used the TCGA genomic data involving 10,975 genes from 80 UM patients [148] and performed weighted gene co-expression network analysis (WGCNA) [156,157,158], a popular method frequently employed to ascertain the potential interactions between genes and phenotypes, which has been successfully utilized in studies in neuroscience [159,160,161], cancer [162,163,164,165] and more recently in COVID-19-applied research [166], among other fields [167]. In a simplistic manner, the WGCNA approach transforms the data of gene expression into modules of co-expression, allowing a better understanding of potential signalling pathways that might be strongly linked with phenotypes of interest [158,167]. It is speculated that WGCNA has the important advantage to correlate co-expression modules with clinically relevant traits, perhaps leading to results with a more meaningful biological significance [158,167]. This robust data analysis methodology has also been employed in metabolomics, proteomics and lipidomics studies [168,169]. In the study conducted by Wan et al., using the TCGA data, their WGCNA analysis yielded 21 different and relevant co-expression gene modules in UM [148]. Out of these 21 co-expression modules, four were demonstrated to be correlated with life status of the UM patient, recurrence and recurrence time [148]. The four distinct hub genes identified were *ABTB1*, *ADPRHL1*, *NTRK2* and *SLC17A7* [148]. Given that the four hub genes were basically oncogenes (*NTRK2*) and genes involved in tumour suppressing pathways (*ABTB1*, *ADPRHL1* and *SLC17A7*), the authors speculated that they might play a vital role in UM reappearance and, thus, constitute important diagnostic and prognostic markers worth studying for UM recurrence detection [148].

In 2019, Xue et al. used the TCGA genomic data to identify a gene signature that could accurately predict the prognosis of UM patients through a methodology involving glmnet COX model and COX regression analysis [149]. After initially identifying 4388 genes with significant prognostic significance in the 80 UM samples included in the TCGA cohort, they developed a robust model involving 18 genes (*AC010442.3*, *AC023790.2, AC092821.1*, *AL137784.1*, *CA12*, *FABP5P1*, *FAM189A2*, *GRIN2A*, *MGLL, MIR4655*, *MMP9, PARP8*, *RNF208*, *S100A13*, *SIRT3*, *TCTN1*, *ZBED1* and *ZNF497*), which allowed the early identification of UM patients with poor and good prognosis [149]. The Kaplan–Meier overall survival (OS) curves of the 18 selected genes prognostic genes showed that high expression of *AC092821.1, FAM189A2, RNF208, SIRT3, TCTN1, ZBED1* and *ZNF497*, as well as lower expression of the genes *AC010442.3*, *AC023790.2, AL137784.1, CA12*, *FABP5P1*, *GRIN2A*, *MGLL, MIR4655,* PARP8 and S100A13 were positively associated with OS in UM patients; meanwhile, the MMP9 expression levels had no significant influence in the survival of poor and good prognosis patients [149]. Later, the Gene Set Enrichment Analysis (GSEA) allowed the identification, among others, of an enriched p53 signalling pathway in the high risk UM group [149], in line with previous pioneer studies establishing that p53 expression in UM cases tends to be correlated with an unfavourable outcome [170].

In 2019, Ni et al. used the TCGA mRNA expression data and performed WGCNA among other complex analytic methods on a group of 5000 genes, which permitted the generation of potential modules involving co-expressed genes and then correlated those modules with clinical and pathological relevant features [150]. The authors were able to find a selection of groups of genes whose expression was associated with tumour-free survival (*ABHD3*, *APOM*, *CALHM2*, *CENPV, CHAC1*, *HTR2B*, *HTRA3*, *LZTFL1*, *UBE2W*, *VCPIP1*, *ZNF391*, *ZNF415*, *ZNF667-AS1 and ZNF835*) and metastasis status (*ABHD3*, *APOM*, *ARFGEF1*, *CALHM2*, *CHAC1*, *CENPV*, *DLL4*, *HTR2B*, *LZTFL1*, *MTUS1*, *NF835*, *SLC25A26*, *UBE2V2*, *UBE2W*, *VCPIP1*, *ZNF391*, *ZNF415* and *ZNF-667-AS1*) [150]. Using a Least Absolute Shrinkage and Selection Operator (LASSO) cox regression model, a 14 validated hub-gene model (*ABHD3*, *APOM*, *CALHM2*, *CENPV*, *CHAC1*, *HTR2B*, *HTRA3*, *LZTFL1*, *UBE2W*, *VCPIP1*, *ZNF391*, *ZNF415*, *ZNF667-AS1* and *ZNF835*) was used to build signatures for prediction of OS and recurrence-free survival (RFS), which were later externally validated using the GEO dataset (GSE27831), in which equivalent results were obtained [150]. The authors demonstrated that in comparison to other robust clinicopathological prognostic parameters, such as TNM classification, chromosomal status or LBD; their 14-gene risk model was superior in predicting OS and RFS [150]. For example, a recent in vitro study involving UM cell lines showed that *CHAC1* downregulation significantly decreased the proliferation and mobility of UM cells [171]. Another interesting piece of data arising from the Ni et al. study was that the KEGG pathway analysis mainly identified pathways related with immune regulation, showing that chromosome 6p gain and chromosome 8q gain, which are associated with reduced UM survival, could have a correlation with a dysfunctional immune system in UM patients, leading to a worse prognosis [150].

In their study, Choi et al. used the TCGA and GEO data (GSE22138 and GSE39717) cohorts and included only patients who died of UM and excluded patients without information on survival status [151]. In a universe of 159 UM patients [TCGA (*n* = 67); GSE22138 (*n* = 63) and GSE39717 (*n* = 29)], the authors used Kaplan–Meier survival analysis with log-rank test to identify genes of prognostic significance that were common among the three distinct cohorts of UM patients [151]. An initial array of 14 genes that had low expression and 37 genes that high expression was identified as being associated with dismal prognosis [151]. Subsequently, a complex protein-protein analysis was performed, demonstrating that three oncogene-like genes (*CYC1, NDUFB9* and *NDUFV2*) and one tumour suppressor-like gene (*CTNNB1*) were main hub genes and significant molecular predictors in UM [151]. Across the three independent cohorts, high expression of *CYC1, NDUFB9* and *NDUFV2*, as well as low expression of *CTNNB1* were systematically associated with decreased survival of UM patients [151]. For example, *CTNNB1* is the gene encoding for β-catenin and the deregulation of the *WNT/CTNNB1* (β-catenin) pathway is a well-established event in the carcinogenic process in several neoplasias, including colorectal cancer [172,173,174], hepatocellular carcinoma [175,176,177] and cutaneous melanoma [178,179,180], among others.

In 2020, Luo et al. used the TCGA cohort gene data of 80 UM patients to develop a 10-gene signature based model for UM prognosis, which was later validated using the GSE22138 data, which includes a group of 63 UM patients [152]. Kaplan–Meier survival analysis and univariate COX regression models were initially employed to screen for genes with prognostic value [152]. Afterwards, COX regression analysis coupled with LASSO methodology was used to achieve the minimum 10-gene prognostic (metastases-free survival) signature, which includes the following genes: *ANXA2P2*, *CA12*, *HMCES*, *POMGNT2*, *RNF208*, *SIRT3*, *SLC44A3*, *STPG1*, *TCTN1* and *ULBP1* [152]. In patients classified has having high-risk score and, thus, shorter survival, they observed a high expression of *ANXA2P2*, *CA12* and *ULBP1* and a low expression of *HMCES*, *POMGNT2*, *RNF208*, *SIRT3*, *SLC44A3*, *STPG1* and *TCTN1* [152]. This 10-gene signature also robustly predicted metastases-free survival (MFS) in the validation GSE22138 cohort [152]. Besides this, the 10-gene risk model was superior in predicting OS when compared to the normally used clinical prognostic parameters, such as TNM classification or LBD [152]. Furthermore, when correlated with UM chromosomal abnormalities, the 10-gene risk model proposed by Luo et al. was shown to have a positive correlation with chromosome 8q copy number and a negative correlation with chromosome 3, 6q and 6p copy numbers [152]. In the high-risk group, the GSEA analysis showed a gene set enrichment in pathways related with immune response, inflammatory response, p53 signalling, proteasome and natural killer cells, among others [152]. This pointed towards a close relationship with tumour microenvironment, which is a theme of increasing interest in UM, given its relevance in UM carcinogenesis and potential therapeutic strategies (please see Section 8) [152].

In 2020, Wan et al. proposed an even more reduced gene signature model for UM prognostication, which encompasses only six genes (*CREG1*, *HIST1H4E*, *LZTS1*, *NIPA1*, *SH2D3A* and *TMEM201*), with multivariate analysis showing it to be a 5-year independent prognostic factor for OS [153]. In brief, the authors used the genomic information of 80 UM patients in the TCGA database and randomly created two datasets [dataset 1 (*n* = 39 patients) and dataset 2 (*n* = 41 patients)] for internal validation [153]. Univariate COX regression analysis allowed the identification of 2010 survival related genes out of a universe of 15,187 genes [153]. Gene functional analysis demonstrated that the identified genes were predominantly connected with mRNA processing, RNA splicing, spliceosome and proteolysis mediated by ubiquitin [153]. A robust likelihood-based survival model methodology was later employed to define the 6-gene signature (*CREG1*, *HIST1H4E*, *LZTS1*, *NIPA1*, *SH2D3A* and *TMEM201*) [153]. High expression of CREG1, HIST1H4E, NIPA1, SH2D3A, as well as low expression of LZTS1 and TMEM201 were demonstrated to be significantly associated with decreased lifetime for UM patients [153]. These results and, thus, the ability of the 6-gene signature to predict 5-year OS in UM patients was also externally validated using two GEO datasets (GSE42656 and GSE84976) [153].

Recently, Tang and Cai generated a model for UM prognosis prediction based on the data of the gene expression microarray GEO data set GSE22138, which comprised a cohort of 63 patients UM patients [154]. Initially, they used WGCNA and identified 41 hub genes that are associated with UM metastases [154]. Afterwards, they applied a LASSO COX regression methodology to identify relevant genes and build a gene expression signature with prognostic significance, which comprises eight genes (*EIF1B, MEGF10, PHLDA1, RPL10A, RPL15, SLC25A38, TFDP2* and *TIPARP*) and named Uveal Melanoma Metastasis Prediction Score (UMPS) [154]. The individual coefficient by LASSO COX regression of RPL10A was demonstrated to be associated with a high risk of metastases, whereas the remainder seven genes were shown to be protective [154]. GSEA analysis showed that the high-risk of metastasis group was associated with complement, E2F targets, G2M checkpoints and unfolded protein response pathways, while no differences in the immune cell proportions were registered between low and high risk groups [154]. The UMPS model was later externally validated using the 80-patient TCGA cohort and the 29-patient GSE27831 cohort of UM patients [154]. The eight-gene expression signature UMPS model was not only able to predict MFS, but was also able to significantly increase the 3-year and 5-year disease-free survival (DFS) prediction accuracy of currently established clinical predictors, such as the AJCC TNM staging [154].

Finally, in a recently published study, Jun Liu et al. proposed a novel six-gene based signature (*ARPC1B, BTBD6, GUSB, KRTCAP2, RHBDD3* and *SLC39A4*) for survival prediction and risk stratification in UM [155]. In brief, using the TCGA database, they initially found that glycolysis and immune response were the most relevant hallmarks for UM related survival [155]. Subsequently, they employed WGCNA, Cox regression analyses and a LASSO algorithm to identify significant hub genes related to glycolysis and immune response, which were used to build the risk model to predict OS of UM patients [155]. The TCGA database constituted the training dataset, while the GEO databases GSE22138 and GSE84976 were used to validate the newly developed prognostic model [155]. Survival analysis demonstrated that the OS of the group with high glycolysis and high immune response Z-scores was lower comparatively to the group with low glycolysis and low immune response Z-scores, respectively [155]. Regarding the immune profile, a higher infiltration of B cells, CD4+ T cells and monocytes was evident in the low-risk group, while the high-risk group had high infiltration by M2-macrophages and myeloid dendritic cells [155]. Their six-gene signature was shown to be an independent and robust prognostic predictor of OS for UM patients [155]. Indeed, ROC curve analysis revealed an AUC above 0.9 for 5-year survival prediction, further validating the six-gene signature as a good model for forecasting the survival of UM patients [155]. Albeit using only six genes, the model was demonstrated to be non-inferior to the 10-gene signature developed by Luo et al. described above for predicting OS [155]. A nomogram based on the six-gene signature was established and might constitute soon, after rigorous validation, a useful tool to develop a personalized therapeutic approach for UM patients [155].

## 6. Immunohistochemistry-Based Novel Prognostic Biomarkers in Uveal Melanoma

Immunohistochemistry is a powerful laboratory technique that has revolutionized Anatomic Pathology over the past decades [181,182]. It is a relatively affordable method to evaluate protein expression and it is readily available and reproducible in most laboratories worldwide [182]. Similarly to other fields within Pathology, researchers in Ocular Pathology have aimed to find protein markers that can be studied through IHC and that can be of prognostic relevance: as previously mentioned, BAP1 is a good example of such marker [140,183]. In the past three years, approximately 20 novel IHC-based prognostic biomarkers in UM have emerged in the literature (Table 4). A review of these biomarkers and respective research in UM is presented below (Table 4).

ATP-binding cassette sub-family B member 5 (ABCB5) is a P-glycoprotein actively engaged in the transport of a myriad of molecules across membranes, including anti-neoplastic molecules [203,204]. ABCB5 is a marker of cancer stem cells and its expression was found increased in different types of neoplasias, including colon cancer [205], cutaneous melanoma [206,207], hepatocellular carcinoma [208] and Merkel cell carcinoma [209]. It is a molecule demonstrated to be implicated in the neoplastic transformation process, tumour expansion and invasiveness [205,207]. For example, in cutaneous melanoma ABCB5 was shown to promote neoplastic invasion and distant metastases through the NF-kB pathway, in a process likely mediated through MMP9, which is involved in cancer invasion and metastasis [207]. In addition, ABCB5 is involved in processes that lead to the resistance of cancer cells to anti-neoplastic agents [204,210]. The expression of ABCB5 was recently evaluated in 32 primary UM cases without associated metastases and 23 primary UM cases with metastases [184]. A higher expression of ABCB5 was observed in the primary UM cases associated with metastases and the authors also showed that these higher levels were correlated with a shorter time to metastases development and, thus, a worse prognosis [184]. Future studies are needed to better understand the role of ABCB5 in UM, its prognostic value and its potential as a therapeutic target.

In the past couple of years, a great interest has been devoted to the expression of Adiponectin in UM, which is a hormone encoded by a gene in chromosome 3, possessing anti-carcinogenic and insulin-sensitizing actions [185]. Tura et al. showed recently that immunoreactivity of Adiponectin and its receptor Adipor1 was decreased in UM cases with M3, suggesting that the lower levels of adiponectin could boost the metastatic potential of UM with that chromosomal abnormality and curb tumour dormancy [185]. Ultimately, adiponectin could be used as a prognostic marker in UM and a potential increase in serum adiponectin levels could be explored as a possible therapy to delay the onset of metastases in UM patients [185]. Interestingly, there was no difference in BAP1 expression between UM cases with low or high levels of Adiponectin and Adipor1 [185].

The role of DDR protein machinery in UM pathobiology remains to be established. The nuclear expression of the ataxia telangiectasia and Rad3-related (ATR) protein, a member of the family of DDR proteins which is encoded in a gene in chromosome 3, similarly to BAP1, was recently evaluated in 69 UM cases [186]. A loss of nuclear ATR expression was documented in nearly 75% of the cases, which was associated with an epithelioid UM cell morphology, increased tumour thickness, increased number of mitotic figures and loss of nuclear BAP1 expression, which are all well-established markers of poor prognosis in UM (Table 2) [186]. This led the authors to conclude that ATR could constitute a novel potential prognostic marker and therapeutic target in UM [186].

Another DDR protein whose expression was recently evaluated in UM is ataxia-telangiectasia mutated (ATM) protein, in a study conducted in 69 UM samples [187]. A loss of nuclear expression of ATM was observed in nearly 65% of the cases and it was significantly correlated with an epithelioid morphology of the UM cells, large tumour diameter above 10 mm, presence of TILs and nuclear BAP1 loss [187]. In addition, patients with absence of nuclear ATM expression had a significant shorter DFS, suggesting that nuclear ATM could constitute a novel biomarker of increased metastatic risk in UM [187]. The correlation between nuclear ATM expression loss with shorter OS could not be established since death was only documented in 2 out of the 69 patients involved in the study [187]. The loss of ATM expression has been observed in other cancer types, namely, breast [211], colon [212] or lung cancers [213], being a strong indicator of a dismal prognosis. Interestingly, the TCGA study showed that DDR proteins were in general upregulated in UM cases with M3 and BAP1 mutations, comparatively to cases preserving two copies of chromosome 3 and harbouring SF3B1 mutations [40].

Autophagy is a natural, homeostatic and complex multi-step cellular process through which the cell eliminates dysfunctional or superfluous components, including lipids, nucleic acids, proteins or organelles, through a lysosome-dependent regulated mechanism [214,215]. Thus, it is fundamental for the orderly degradation and recycling of cellular components, being instrumental for adequate cellular differentiation and survival, as well as tissue development [214,215,216]. Autophagy has a dual role in cancer, since it is important in tumour suppression in early states of the neoplastic development process, while in more advanced neoplastic states it is upregulated leading to a pro-survival and tumourigenic effect in neoplastic cells, enhanced proliferation and metastases [216,217,218]. The role of autophagy in UM development is poorly understood. Recently, the expression of three proteins [autophagy-related gene 7 (ATG7), Beclin-1 and p62] belonging to the vast family of proteins involved in autophagy was assessed through IHC in a cohort of 85 cases of primary UM [188]. Higher expression of Beclin-1 was correlated with a decreased risk of metastases and extended DFS times, establishing Beclin-1 as a significant positive prognostic factor in UM [188]. Contrarily, the expression of ATG7 and p62 did not impact significantly on the prognosis of UM patients [188]. Together, these results open novel avenues towards the evaluation of autophagy-related molecules as prognostic factors in UM and also as potential innovative therapeutic strategies.

BCL2 19 kD protein-interacting protein 3 (BNIP3) is a mitochondrial protein belonging to the BCL-2 family, which has been demonstrated to be involved in the complex regulation of cell death, autophagy and cellular protection [219,220]. Regulation of BNIP3 levels has been implicated in different types of neoplasias, namely, breast cancer [221], lung cancer [222], salivary adenoid cystic carcinoma [223] and skin melanoma [224], being associated with progression of the disease and prognosis. In a recent study, the expression of BNIP3 was evaluated through IHC in a cohort of 47 primary UM cases and the authors demonstrated that higher levels of BNIP3 were correlated with a shorter survival [189]. Given that BNIP3 has both cell death and cell survival promoting effects, novel studies will be needed to elucidate the role of this marker in UM.

Butyrophilin (BTN) and butyrophilin-like (BTNL) family of proteins are structurally related with B7-molecules and like-B7 molecules, being all critical in the modulation of T-cell mediated immune function [225,226]. Even though our knowledge on the regulation of T-cells by BTN and BTNL proteins is still scarce, they appear to be involved in inflammatory diseases and cancer [225,226]. The mRNA expression of BTNL9, one member of the family, was demonstrated to be low in colon cancer comparatively to normal colon [225]. In addition, in a recent study, researchers took advantage of the TCGA database and verified that the expression of BTNL9 was downregulated in breast cancer [226]. The lower expression of BTNL9 in breast cancer was significantly correlated with a worse DFS and OS [226]. Later, by studying breast cancer cell lines they demonstrated that BTNL9 might have an anti-cancer role in breast cancer by inhibiting proliferation and metastasis [226]. A more recent study on breast cancer, involving a multiomics approach, also showed that higher mRNA levels of BTNL9 and of other family members in the BTN/BTNL family were associated with a more favourable DFS and extended OS [227]. The expression of BTNL9 was also recently evaluated in a cohort of 62 primary UM cases [190]. A higher expression of BTNL9 was significantly correlated with a better OS, suggesting that BTNL9 is a marker of good prognosis in UM [190]. This study has opened the prospect of modulating BTNL9 expression as a possible therapeutic option for UM. Comprehensive studies are needed to better understand the role of BTNL9 expression in UM, as well as the possible interplay between BTNL9 expression in UM cells and immune regulation.

The expression of Ephrin receptors has been studied in different tumours with data indicating that they might have an important role as prognostic factors [228,229]. They constitute the largest known subfamily of receptor tyrosine kinases (RTKs), which play critical roles during the embryonic development, such as axon guidance, cell migration, segmentation and formation of tissue boundaries [230,231,232,233]. In addition, during adulthood, they have roles in angiogenesis, stem cell differentiation, immune system regulation and in cancer development, among others [228,229]. Our knowledge on the expression of Ephrin receptors in UM expanded recently with a study where the expression of EphA1, EphA5 and EphA7 was evaluated in 94 UM enucleation samples without previous treatment [192]. A decreased expression of EphA1 and EphA5 was associated with a worse prognosis for UM patients, while a prognostic role could not be firmly established for EphA7 expression [192]. Indeed, a smaller tumour size, decreased mitotic activity and absence of extrascleral extension were positively correlated with increased EphA1 expression, whereas higher EphA5 expression was linked to absence of metastases and decreased likelihood of chromosome 3 loss [192]. This study established that EphA1 and EphA5 are potentially important prognostic markers in UM patients and also opened the prospect of using small molecules addressing the Eph/ephrin signalling as candidate therapies for UM [192,234].

Histone Deacetylases (HDACs) are known to have fundamental roles in the regulation of cellular proliferation, differentiation, angiogenesis and cell death, being implicated in neurodegeneration [235,236] and different forms of cancer, including lung cancer [237,238], skin melanoma [239] and lymphoma [240], among others. Consequently, HDAC inhibitors have constituted promising anti-neurodegeneration and anti-cancer therapies [241,242]. The prognostic significance of HDAC expression in UM was recently evaluated in a study involving 75 UM cases [193]. In line with previous studies, which confirmed HDAC gene [243,244] and protein [245] expression in UM, the authors evaluated HDAC-1, HDAC-2, HDAC-4 and HDAC-6 through IHC and aimed to determine their role as prognostic factors [193]. HDAC-1 and HDAC-2 had both nuclear and cytoplasmic expression, whereas HDAC-4 and HDAC-6 were mostly expressed in the cytoplasm of UM cells [193]. Among the four studied HDACs isoforms, HDAC-2 was the most frequently expressed, with a more significant nuclear expression pattern, and the expression of HDAC-2 the only proven to be an independent factor of better survival in UM [193]. This study provides additional evidence on the potential role of HDACs in UM development and progression, suggesting that inhibition of HDAC could constitute a relevant therapeutic strategy [241]. In a recent phase 2 clinical trial involving 28 patients with metastatic UM, Entinostat (HDAC inhibitor small molecule) was tested combined with Pembrolizumab (inhibitor of PD-1) [246]. Encouraging positive responses in terms of progression-free survival (PFS) and OS were observed in a well-defined subset of mestastic UM patients, namely, patients with BAP1-preserved tumours and one patient with iris melanoma containing a UV-related gene signature [246]. There is an ongoing phase 2 clinical trial in metastatic UM involving the HDAC inhibitor Vorinostat as monotherapy (ClinicalTrials.gov: NCT01587352), which will give us important pilot data on the efficacy of this therapeutic avenue in UM.

Nestin is a well-known intermediate filament protein family member, constituting a putative marker of stem cells in the CNS [247,248], an established cancer stem cell marker [249,250] and a prognostic marker in different tumours, including breast cancer [251], colorectal cancer [252] and lung cancer [253], among others. In fact, the increased expression of nestin in these tumours was associated with an immature stem-cell like phenotype, chemoresistance and enhanced capacity for invasiveness [250,254]. Recently, the team of Sarah Coupland studied the expression of nestin in 141 cases of primary UM and found a correlation between nestin positivity (defined as expression above a cut-off value of ≥10% positively stained UM cells) and well-established factors of bad prognosis in UM, such as epithelioid morphology, higher mitotic counts, M3 and chromosome 8q gain [194]. Besides this, the Kaplan–Meier survival analysis also confirmed that primary UM cases displaying nestin positivity had a worse survival comparatively to nestin-negative cases [194], a finding that has also been corroborated by nestin expression analysis in the TCGA cohort by the same research group [255]. The expression of nestin in UM metastases was also studied and expression of the marker was consistently found in nearly 80% of the cases [194]. Interestingly, the expression of nestin was not observed in the normal choroidal melanocytes, which suggests that in line with other cancer types, the tumourigenic process in UM might involve transformation into a more immature/stem-cell-like phenotype [194]. Together, these results show that high expression of nestin is associated with a more aggressive UM phenotype, displaying enhanced capacity for development of metastases and a significantly decreased survival after diagnosis [194].

The NF-κB (nuclear factor kappa-light-chain-enhancer of activated B cells) comprises a family of transcription factors that finely regulate a large array of genes involved in cell survival, inflammatory disorders, response to infection, autoimmune disorders and cancer, among other processes [256,257,258]. Five main structurally related members compose that family: p50 (also named NF-κB1), p52 (also named NF-κB2), p65 (also named RelA), RelB and c-Rel [257,258]. A family of inhibitory proteins normally sequesters the different NF-κB proteins in the cytoplasm [257,258]. The adequate activation of the major NF-κB pathway involves two major signalling pathways, the canonical and non-canonical (or alternative) pathways, both being instrumental to regulate the cellular processes governed by NF-κB [256,259]. In the past two decades our knowledge on the NF-κB pathway has considerably expanded, positioning this pathway as an instrumental orchestrator in the regulation of inflammation and in the development of different tumours, including UM [257,260,261]. Using IHC, in 75 UM cases, Singh et al. evaluated the expression of p52 and RelB members of the NF-κB family [196]. They showed that the expression of p52 and RelB was associated with BAP1 loss [196]. Furthermore, in metastatic cases with LBD above 15 mm, tumour thickness exceeding 8 mm and higher tumour staging, the expression of p52 and RelB was significantly increased [196]. The MFS time was decreased in cases positive for p52, RelB, and p52/RelB co-expression [196]. Cases with higher p52 expression and p52/RelB co-expression had worst OS [196]. In another study, they evaluated the expression of three members of the canonical NF-κB pathway in UM: p50, p65 and c-Rel [195]. Nuclear immunoreactivity of p65, p50, and c-Rel significantly correlated with well-established prognostic factors, such as, LBD > 12 mm, tumour height > 8 mm, microvascular density, TILs, TIMs and, more importantly, metastases development [195]. The presence of nuclear p50 and p65 immunorreactivity was associated with a lower survival for UM patients, while the expression of c-Rel was not shown to impact on the OS [195]. The multivariate analysis later established nuclear p50 and p65 expression as independent UM prognostic factors [195]. Interestingly, in a subsequent study, the expression of c-Rel was assessed in 75 UM cases [191]. Nuclear expression of the c-Rel protein, which suggests NF-κB activation, was observed in 56% of the studied UM cases [191]. The nuclear expression of c-Rel was significantly correlated with an epithelioid UM cell morphology, invasion of the ciliary body and iris, as well as scleral invasion [191]. In line with this, patients with nuclear c-Rel expression had an inferior survival [191]. Together, these results show that different proteins in the NF-κB pathway can constitute novel prognostic factors in UM and that promising novel anti-neoplastic approaches in UM might be explored through regulation of this pathway.

Poly(ADP-ribose) polymerases (PARP) constitute an important protein family with fundamental roles in different cellular processes, including DNA repair and programmed cell death, being implicated in cancer development and therapy [262,263]. One of the most extensively studied members of this family is PARP-1, whose expression in UM was recently evaluated through IHC in a study involving 91 enucleation samples [197]. An increased tumour size, higher histopathological grade and higher chromosome 3 loss frequency were significantly correlated with increased expression of PARP-1 [197]. Furthermore, smaller DFS and OS were associated with higher expression of PARP-1, suggesting that this protein could constitute a novel relevant biomarker of poor prognosis in UM [197] and that PARP inhibitory therapies could be evaluated in UM treatment [264].

Immune checkpoint inhibitor therapies have successfully revolutionized the landscape of therapeutic weapons in different types of cancer, namely, lung cancer [265], skin melanoma [266], head and neck cancer [267], among others, and is being envisioned as one of the current most promising strategies to tackle cancer. Programmed cell death receptor-1 (PD-1), one of the best studied and most advanced immune checkpoint inhibition targets, is expressed in lymphocytes (T and B cells), macrophages and natural killer cells and their effector function is halted when it binds its co-ligands programmed cell death-ligand 1 (PD-L1) and programmed cell death-ligand 2 (PD-L2), which are expressed in contacting cells, including antigen-presenting cells, regulatory T-cells and neoplastic cells [268,269]. However, the expression of PD-1 has also been documented in human melanoma cells, even in the absence of a tumour microenvironment, leading to enhancement of tumour growth [270]. Our understanding of the role of immunotherapy in UM is still in its infancy, especially since the blockade of the PD-1/PD-L1 axis has not yielded significant results in this type of tumour [271,272,273] (please see Section 8). The expression of PD-1 in UM was recently evaluated through IHC in a tissue microarray cohort of 82 primary UM cases [198]. Patients with a high expression of PD-1 in the tumoural cells had a smaller DFS and decreased OS [198]. In the same study, the author overexpressed PD-1 in UM cell lines and found that cells had an enhanced proliferative capacity, which was halted when PD-1 expression was downregulated using shRNA [198]. However, in the hands of others [199] and in our hands (data not shown), PD-1 expression in primary UM cases, through IHC, has not been observed, being only present in infiltrating inflammatory cells. In a subsequent study, Singh et al. evaluated the expression of PD-1 and PD-L1 in 71 UM cases [199]. They observed expression of PD-1 in TILs in 30/71 cases, while PD-L1 was expressed mostly in UM cells in 44/71 cases [199]. In the multivariate analysis, PD-1 and PD-L1 immunoexpression were shown to be significant prognostic factors of a reduced DFS [199]. However, in this cohort, patients without TILs displaying PD-L1 expression had an extended DFS and, thus, a better prognosis [199]. This data is in accordance with an earlier study involving 67 primary UM cases, in which PD-L1 expression in more than 5% of tumoural cells was associated with lower number of TILs and a lengthier MFS [274]. Interestingly, expression of PD-L1 in immune infiltrating cells did not significantly impact on the prognosis of the studied cohort [274]. On the multivariate analysis, the UM patient sub-group with PD-L1 expression in more than 5% of UM cells and in immune cells infiltrating the UM had a longer period free of metastasis and, thus, a less adverse prognosis [274]. These results suggest that PD-L1 expression in UM could signal a more positive outcome [274]. Novel studies are needed to further elucidate the role of PD-1/PD-L1 axis in UM development and metastization, as well as to optimize supplementary strategies to turn checkpoint inhibition into an effective therapeutic strategy for UM (please see Section 8).

Polio-like kinase 1 (PLK-1) expression in UM was also recently reported [200]. PLK-1 is a conserved kinase mainly involved in the regulation of cell cycle [275,276,277] and increased expression of PLK-1 was described in breast cancer [278], lung cancer [279] and lymphoma [280], among others, and is being considered a potential molecular target in anti-cancer therapies [281,282]. Berus et al. performed IHC for PLK-1 in 158 UM cases and found that 30% of the tumours had low expression of PLK-1, which was correlated with a higher TNM staging and, thus, a significantly decreased OS [200]. A firm correlation between PLK-1 levels and DFS could not be determined in this study [200]. Even though, contrarily to what was described in other tumours, lower expression of PLK-1 seems to point towards a decreased life expectancy in UM patients [200].

The thioredoxin-dependent peroxidase reductase (PRDX3) is an enzyme localized in the mitochondria which has a fundamental role in the antioxidant defence of cells [283,284]. The enhanced expression of PRDX3 has been reported in different types of cancers [283], but its expression in UM was only recently unravelled [201]. PRDX3 IHC was performed in tissue microarrays of 92 UM samples. A significant strong correlation between high PRDX3 expression and metastatic disease development and reduced OS was demonstrated, proposing that high PRDX3 expression in UM is also a marker of poor prognosis [201].

Sperm proteins associated with the nucleus on the X chromosome (SPANX) family members (SPANX-A, -B, -C and -D) are normally expressed in the testis during spermatogenesis [285,286,287]. Interestingly, numerous studies also demonstrated the involvement of SPANX proteins in cancer development, namely, in breast cancer [288] and cutaneous melanoma [289,290], among others. The role of this protein in cancer development remains to be firmly understood, but the available studies suggest a role in promoting cancer growth and invasiveness [288,289,290]. The expression of SPANX-C was also recently evaluated in 55 primary UM cases [202]. The research team demonstrated a higher expression of SPANX-C in UM that had developed metastases [202]. In addition, patients with a higher expression of SPANX-C in the primary tumour had a decreased MFS [202]. Thus, higher levels of SPANX-C in UM could constitute a new marker of dismal prognosis [202].

## 7. Additional Novel Promising Molecular Biomarkers in Uveal Melanoma with Prognostic Relevance

Comprehensive proteomic analysis has started to contribute to better define the prognosis of UM patients. The largest study involving proteomic analysis of primary UM patients using a LC MS/MS iTRAQ methodology was unveiled recently, involving 53 metastasizing and 47 non-metastasizing cases [291]. In all studied cases, nearly 3935 different proteins were evaluated and bioinformatics analyses allowed the identification of 191 differentially expressed proteins elevated in metastatic cases and 211 differentially expressed proteins elevated in non-metastatic cases [291]. Reactome pathway analysis of proteins preferentially elevated in metastatic UM showed mostly an overrepresentation of immune system pathways, but also pathways associated with vesicle-mediated trafficking, extracellular matrix organization, metabolism of proteins and homeostasis [291]. On the other hand, in non-metastatic cases, the authors demonstrated a preponderance of pathways connected with metabolism, but also cellular response to external stimuli and developmental biology [291]. Interestingly, the over-representation of proteins of the immune system pathways was more relevant in metastatic cases, while housekeeping pathways were over-represented in non-metastatic cases [291]. This study helped to highlight the immune suppressive nature in primary UM, demonstrating a rather low abundance of immune checkpoint regulator molecules [291]. Yet, some molecules, like CDH1 and HLA-DPA1, as well as 15 kinases and phosphatases emerged as novel candidates for immune checkpoint inhibition therapies [291]. Finally, the authors developed a robust model incorporating 32 proteins which was able to predict metastases development with a 93% discriminatory accuracy [291]. Interestingly, studies like this can lead to the development of innovative immunoassays for the non-invasive UM diagnosis using blood or other biological samples [292,293,294]. Ensuing studies involving larger cohorts of UM patients will be of fundamental value to better define the potential of prognostic protein profiles based on proteomics of primary UM cases.

## 8. Current Challenges and Future Perspectives in Uveal Melanoma

The comprehensive work carried out in the field of Ocular Oncology over the past years has considerably increased our knowledge on Uveal Melanoma. However, our capacity to prevent UM metastization and UM-related death has not changed considerably over the past years [9,48]. Below, we highlight the most relevant current challenges in UM and anticipate some of the future avenues in UM research and UM patient management.

### 8.1. Need for Accurate and Robust Models for Prognostication in Uveal Melanoma

In uveal melanoma research, most reported studies involve a limited collection of patient samples, as it became evident, for example, in the description of the novel IHC-based markers that have been reported recently. In addition, the methodology of analysis is also rich and heterogeneous, as it was observed in the recent efforts to find gene signatures with prognostic relevance in UM. Therefore, this poses enormous challenges in the validation and generalization of the obtained results for a wider community of UM patients. One approach could be the stimulation of the widespread sharing of pre-clinical and clinical data, similarly to the history of The Collaborative Ocular Melanoma Study (COMS) initiative, which yielded impactful and groundbreaking contributions to field [295]. The assembly of comprehensive UM patient databases through collaborative efforts could significantly and positively impact on UM research. Indeed, taking advantage of large clinical databases, together with genetic information and pre-clinical data, coupled with artificial intelligence (AI)-based strategies, it will be possible to develop novel robust artificial neural network-based systems to confidently predict patient survival and stratify UM patients for follow-up, therapy and possibly enrolment in clinical trials. Ultimately, this has the prospect to lead to the long-aimed development of a model of UM patient prognostication that is widely accepted and employed by the UM community worldwide. In line with this, among the first successful tools developed is the Liverpool Uveal Melanoma Prognosticator Online (LUMPO), a bioinformatic tool established from the data gathered from patients in the United Kingdom, assembling clinical, histological and genetic data [143,296]. LUMPO is available online, allowing a reliable prognostication of individual UM patients through determination of the risk of metastases and estimation of survival time [143,296]. A subsequent web-based tool created for UM patient prognostication is the Prediction of Risk of Metastasis in Uveal Melanoma (PRiMeUM), established from data obtained mainly from UM patients in the United States of America (USA) [297]. PRiMeUM allows a reliable personalized determination of the metastatic risk at 48 months post-initial diagnosis by combining clinical features (age, sex, tumour location, LBD and TT) and detailed information on chromosomal analysis (chromosome 1p, 3, 6p, 6q, 8p and 8q status) [297]. Even though both tools are able to ascertain the metastatic risk of patients, none can determine accurately when the metastases will develop [143,296,297].

### 8.2. The Promise of Liquid Biopsies for Uveal Melanoma

Liquid biopsies (LBs) have opened unprecedented avenues in the field of cancer, by allowing a non-invasive approach for diagnosis, identification of relevant mutations, disease progression monitoring, early disease recurrence detection and evaluation of response to therapies, among others [298,299,300]. They are based on the testing of blood or other body fluids (for example, the aqueous humour) and constitute an alternative reproducible method to the classical tissue biopsy, which can be used to detect circulating tumour DNA (ctDNA), circulating tumour cells (CTCs), exosomes, cytokines and microRNAs, among other components [298,300,301]. LBs have been particularly promising in different solid tumours, including lung cancer, with some studies demonstrating encouraging results in their usage in the daily practice [302,303], despite some current technical limitations [304,305,306]. In UM, the LB technology is not yet as developed as it is for other types of cancers and there are no currently available LB systems approved by both the European Medicines Agency (EMA) and Food and Drug Administration (FDA) [298]. However, there is a hope that LBs will become a solid alternative to intraocular biopsies, avoiding the risks of the procedure, including possible tumour dissemination [298,307,308,309]. For example, in a recent published study including 21 patients with UM metastatic disease in a cohort of 135 UM patients, the authors showed that ctDNA was detectable in the plasma of 17 of the 21 metastatic patients [310]. More importantly, by analysing GNAQ/GNA11 mutations using deep amplicon sequencing, in 10 of those UM patients the detection of ctDNA occurred at least 2 months up to 10 months before the clinical detection of metastases, further emphasizing the extraordinary potential of this innovative diagnostic methodology [310].

Previous studies revealed that in UM an inflammatory microenvironment, including infiltration by lymphocytes and macrophages, portends a bad prognosis for patients [122,311]. Knowing that a myriad of proteins can be robustly identified in the anterior chamber fluid and vitreous, Wierenga et al. recently evaluated whether the aqueous humour could be used to measure cytokines and, consequently, define cytokine profiles that could establish an accurate prognosis for UM patients [312]. The analysis of the aqueous humour as a substitute of tumour biopsy has been previous successfully employed in cases of retinoblastoma for diagnosis using cell free DNA [313,314], as well as for detecting the expression of IL-10 and IL-6 as biomarkers for the diagnosis of intraocular lymphoma [315,316]. The study conducted by Wierenga et al. involved 84 UM enucleation samples, from which aqueous humour was immediately collected after surgery [312]. The Proximity Extension Assay (PEA) technology that was employed allowed the detection of 92 proteins using only 1 µL of sample [312]. The study of 84 cytokines in the aqueous humour which were consistently above the limits of detection of the assay, led to the definition of three main clusters: a cluster with few cytokines (cluster 1; *n* = 37), a cluster with an intermediate number of cytokines expressed (cluster 2; *n* = 36) and a cluster enriched in several cytokines (cluster 3; *n* = 11) [312]. Adenosine deaminase (ADA), CD244, CD40, galactin-9 (Gal-9), monocyte-chemotactic protein 3 (MCP-3), PD-L1, tumour necrosis factor receptor superfamily 21 (TNFRSF21) and tumour necrosis factor-related apoptosis inducing ligand (TRAIL) were the most differentially expressed cytokines among the three defined clusters [312]. High levels of CD40, Gal-9, tumour necrosis factor receptor superfamily 9 (TNFRSF9), TNFRSF21 and Fas Ligand (FASLG) were registered in the aqueous humour of patients who had worst survival [312]. Interestingly, the majority of these cytokines are involved in the regulation/induction of apoptosis [312]. When the authors correlated the clinicopathological data with the cytokine cluster analysis, they observed that clusters 2 and 3 were associated with worse prognostic features [312]. Interestingly, cluster 1 patients were shown to have better survival than cluster 2 and cluster 3 patients, while there were no significant survival differences between clusters 2 and 3 patients [312]. Interestingly, in another study involving a cohort of 35 UM patients, the authors found significant higher levels of interleukin-6 (IL-6), interleukin-8 (IL-8), regulated upon activation normal T cell expressed and secreted (RANTES), epidermal growth factor (EGF), basic fibroblast growth factor (bFGF), macrophage migration inhibitory factor (MIF) and monocyte chemoattractant protein-1 (MCP-1) in the aqueous humour of UM patients when compared to control patients [317]. A positive correlation between IL-6 levels and the degree of retinal detachment, as well as between IL-8 levels and tumour thickness was established, which proposes that the levels of these cytokines could hint a more advanced disease stage [317]. Survival analysis was not performed; however, the described pro-inflammatory environment could be linked with an enhanced tumour growth and infiltration by immune cells, which are both associated with a poor survival [317]. Comprehensive studies, with larger cohorts of UM, will be instrumental to define prognostic profiles based on biomarkers present in the aqueous humour. Altogether, these pioneer studies demonstrate that the continuous development in the technological procedures employed in LBs has the potential to soon critically change the paradigm of UM diagnosis and follow-up.

### 8.3. The Relevance of Non-Coding RNAs (ncRNAs) in Uveal Melanoma

Non-coding RNAs (ncRNAs), which comprise a vast family of very small molecules, including long non-coding RNAs (lncRNAs) and microRNAs (miRNAs), influence and regulate genes at the post-transcriptional level, being involved in different cell types in the fine-tuned control of cell proliferation, cell differentiation and cell death, among other processes [318,319]. Similarly to other cancers, such as lung cancer [320] or cutaneous melanoma [321], in UM ncRNAs constitute emerging molecular players given that aberrations in their expression have been implicated in the development and progression of the disease [322,323]. For example, miRNAs were shown to have both tumour-suppressing and tumour-promoting roles, which are being increasingly unveiled [324,325]. Researchers have tried to uncover miRNAs differentially expressed in cases of primary UM with low and high risk of metastases, with a recent research pooling results from different studies showing consistently in the high-metastatic risk group upregulation of the miRNAs let-7b, miR-20a, miR-124, miR-142, miR-155, miR-199 and miR-224, while only miR-181a and miR-211 were constantly downregulated [324]. The MAPK and PI3K-Akt signaling pathways appear to be altered in light of the dysregulation of the different collection of miRNAs in UM [324,326]. Besides constituting biomarkers of the disease, miRNAs could also become soon promising therapeutic targets in metastatic UM [326,327,328]. Larger studies, involving well-established methodologies will be of fundamental importance to institute meaningful signatures of ncRNAs in UM.

### 8.4. Dissecting the Role of Tumour Infiltrating Immune Cells in Uveal Melanoma

Mounting evidence points towards a unique biological behaviour in UM, with infiltration by immune cells strongly linked with a tumour growth stimulatory effect, instead of a tumour suppressing effect, which is normally present in other types of neoplasias [272,311,329]. In addition, unlike in cutaneous melanoma, immune checkpoint inhibition has not yielded meaningful results for the majority of UM patients [123,330,331]. Together, these results highlight the presence of a strongly immunosuppressive microenvironment in primary UM, and our knowledge on this distinctive phenomenon remains in its infancy [329,332,333]. Interestingly, an association between BAP1 loss and the infiltration of the tumoural microenvironment by lymphocytes and macrophages, with concomitant overexpression of genes involved in immunosuppression has been established [332]. Classically and well-established checkpoint inhibitor molecules such as CTLA4 and PD-1 seem to be relevant only for limited subsets of UM patients [123,330,331]. On the other hand, a recent study involving single cell RNA sequencing demonstrated that an emerging checkpoint inhibitor molecule, lymphocyte-activation gene-3 (LAG3), is expressed at high levels in most of T-CD8+ cytotoxic UM TILs [334]. Furthermore, increased expression of LAG3 in UM is linked with M3/BAP1 loss (associated with the highest risk of metastasis development) and strongly correlated with a high metastases rate and a worst survival [335]. The expression of LAG3 was proven to be positively correlated with the expression of several of its ligands, namely, Galectin-3 and several molecules in the HLA class II family [335]. LAG3-expressing lymphocytes were also documented in the liver metastases of UM patients [334]. Thus, LAG-3 could be among the most relevant immune checkpoint molecules in UM and, similarly to cutaneous melanoma, anti-LAG3 directed therapies could soon have a role in the treatment of UM patients [334,335].

Cluster of differentiation 47 (CD47) is a transmembrane integrin associated protein of the immunoglobulin superfamily that acts as a “do not eat me” signal for macrophages through binding to the signal regulatory protein (SIRP) on antigen presenting cells [336,337]. The levels of CD47 are normally decreased in damaged or senescent cells, priming their clearance by macrophages [336,337]. In contrast, CD47 was demonstrated to be overexpressed in different cancer types, constituting a strong independent marker of poor prognosis [338,339,340]. The expression of CD47 in UM patients was studied using the TCGA database [341]. Patients with lower CD47 levels had a better PFS, even though there were no major survival differences between patients with low and high CD47 levels [341]. In addition, higher levels of CD47 were associated with a higher immune score, namely, an increase in the number of TILs (CD4+ and CD8+ T cells), proposing that anti-CD47 therapies could also constitute a novel and relevant therapeutic avenue in UM [341,342].

The adequate evaluation of the expression of established (PD-1/PD-L1, CTLA-4) and emerging immune checkpoints molecules [B7 homolog 3 protein (B7-H3), inducible T cell costimulatory (ICOS), indoleamine 2,3-dioxygenase (IDO), LAG3, T cell immunoreceptor with Ig and ITIM domains (TIGIT), T cell immunoglobulin-3 (TIM-3), V-domain Ig suppressor of T cell activation (VISTA), among others] [333,343,344], as well as other immune relevant proteins will be instrumental for the development of novel therapies for UM, which will likely be based on the well-thought combination of different immune blocking antibodies rather than a single miracle antibody approach [273,345]. In this regard, the advent and optimization of laboratory techniques such as multiplexed IHC will critically impact in our ability to identify specific protein markers of interest and/or molecular abnormalities, allowing a more accurate assessment of the in vivo interplay between the different cells of the immune system and tumoural UM cells [346,347].

### 8.5. Unravelling the Mystery of Preferential UM Metastization to the Liver

The leading cause of death in patients with UM is metastization, which preferentially occurs to the liver (Table 1) [24]. Our knowledge on the mechanisms underlying the exquisitely preference of UM cells for the liver microenvironment is still scarce [9]. In the liver, metastasizing UM cells can display two fundamental patterns of growth: nodular periportal or infiltrative sinusoidal [9,348,349]. The periportal nodular growth pattern is characterized by the presence of UM cells concentrated in the periportal areas, which then become hypoxic, leading to angiogenesis promoted by vascular endothelial growth factor (VEGF), which is produced by the neoplastic cells [348,349]. On the other hand, the infiltrative pattern is characterized by UM cells that occupy the sinusoidal spaces, with a lesser degree of hypoxia and that also develop their own circulation through a process which involves hepatic stellate cells [348,349]. The expression of cMET by UM cells is a plausible explanation for their preferential homing to the liver, since hepatic stellate cells produce the hepatocyte growth factor (HGF), the ligand of cMET [9,350]. Similarly, UM cells also express CXCR4 and hepatic sinusoidal endothelial cells and hepatic stellate cells produce its ligand CXCR12 [9,350]. Interestingly, the blockade of both axes in rodent models also prevented UM metastization [9,350]. Mounting evidence also demonstrates that immune related mechanisms are involved in the promotion of growth of UM cells in the liver microenvironment [291,329]. For example, there is a preponderance of M2-TAMs infiltration in UM metastatic to the liver [329]. Interestingly, the metastatic UM cells in the liver upregulate the expression of an array of genes (BCL2, CD44, CD146/MCAM/MUC18, DUSP4, IGF1R, IRF4/MUM1, LGALS3/Galectin-3, MFGE8/lactadherin, PNMA1 and PRAME), which is likely to contribute to an immunosuppressive tumoural microenvironment [329]. In a recent study, the metastatic tissues of a UM patient with liver metastasis were submitted to a comprehensive single-cell RNA sequencing (scRNA-seq) study, which exposed an extensive intra- and inter-tumoural heterogeneity, further highlighting the diversity and complexity of UM even in a single individual [351]. Interestingly, a high degree of intratumoural heterogeneity has also been demonstrated in primary UM [352,353]. A deeper understanding of the mechanisms involved in the development of liver metastases by UM, including the role of intratumoural heterogeneity, will be fundamental for the generation of robust therapeutic strategies aiming at preventing UM metastases and approaches directed to ablate those metastases with the ultimate aim to significantly increase the life expectancy of patients diagnosed with UM [353].

### 8.6. Novel In Vitro Cancer Models Will Likely Boost Research Efforts in Uveal Melanoma

The capacity to culture in vitro cells of human origin has dramatically changed the landscape of medical research over the past decades [354,355]. The first human cell line was the HeLa cell line, established in 1951 from a biopsy sample of Henrietta Lacks, who had an aggressive adenocarcinoma of the cervix [356]. Since then, a myriad of different immortalized cell lines and cell culturing approaches from patient-derived tissue have made possible the study of human cells in vitro, both in simple monolayer cultures and in more complex 3D models. Cell lines have been instrumental to unravel important UM biological mechanisms and also allowed the testing of some drugs [357,358,359]. Even though successful culturing in vitro from primary UM cells has been achieved, a standard procedure remains to be established and different groups use their own approaches [357,358,359,360]. Furthermore, reliable animal models remain to be established [361,362]. One appealing strategy can be the usage of human induced pluripotent cells (hiPSCs, efficiently generated from reprogramming of somatic cells obtained from donors), which similarly to human embryonic stem cells (hESCs), are successfully cultured in vitro and are able to differentiate into all three embryonic germ layers (ectoderm, mesoderm and endoderm) and give rise to virtually all cell types of the body in inexhaustible manners [355,363]. In the case of hiPSCs, they retain the genetic background of the donor [363]. Both hiPSCs and hESCs have allowed the study of human cells which are not normally accessible to study in the human body (for example, neurons and glial cells of the human CNS) and, therefore, have boosted the possibilities in medical research employing human cell lines, permitting the study of mechanisms of human development [363,364]; in vitro disease modelling, including in cancer research [365,366,367]; in the development of assays and platforms for drug screening campaigns [355,368,369]; in patient stratification and in the development of cell replacement strategies [355,363]. The pioneer monolayer cultures gave way to organoids, spheroids, organ-on-a-chip approaches and more recently assembloids, which employ single cell types or a multitude of different cellular types [355,360,370,371,372,373]. One such example is the recently developed model of 3D cortico-motor assembloids by Paşca and collaborators, which has brought this technology into a new state of development [374], allowing the efficient combination of 3D structures analogous to the cerebral cortex and/or the hindbrain/spinal cord with human skeletal muscle spheroids, generating a functional nervous circuit in vitro [374]. These innovative 3D cortico-motor assembloids established the conditions for unprecedented opportunities in terms of disease modelling and drug discovery in motor neuron disorders [374,375]. Hopefully, our increased ability to grow different cell types in vitro will lead in the near future to the development of UM 3D models that capture the organization, tumour microenvironment and cellular milieu of the UM patient. Those models will have far reaching impact in our understanding of the disease, in patient stratification and prognostication, as well as in drug discovery and development. In this respect, for example, models of blood–eye barrier and UM-liver co-cultures will offer unprecedented possibilities.

### 8.7. The Need for Novel Effective Therapeutics for Metastatic Uveal Melanoma

The OS of patients diagnosed with UM has not dramatically changed over the past decades given that there are only limited therapeutic possibilities once widespread metastatic disease develops. In the past year, promising results on the usage of Tebentafusp for UM have been published [376,377]. Tebentafusp is an innovative engineered fusion protein belonging to a new class of promising therapeutic agents termed immune-mobilizing monoclonal T-cell receptors against cancer (ImmTAC) [376,377]. It is a construct composed of a soluble affinity-enhanced HLA-A*02:01–restricted T-cell receptor specific for the glycoprotein 100 (gp100) peptide YLEPGPVTA which is fused to an anti-CD3 single-chain variable fragment [376,378]. Thus, ImmTACs like Tebentafusp, are able to target any protein presented as a peptide–HLA complex on the surface of the target-cell, including intracellular antigens [379,380]. The binding of ImmTAC molecules to their target-cell surface specific peptide–HLA complexes initiates the recruitment and activation of polyclonal T cells, mediated through CD3, which efficiently leads to the release of cytokines and other cytolytic mediators to the target cells [379,380]. In a recent phase 3 clinical trial involving 378 previously untreated HLAA*02:01–positive metastatic UM patients, stratified based on lactate dehydrogenase (LDH) levels, treatment with Tebentafusp was associated with a higher PFS and a 1-year OS of 73%, while in the control group (single agent pembrolizumab, ipilimumab, or dacarbazine) the observed 1-year OS was only 59% [376]. Even though Tebentafusp showed promising results in all UM patients, new studies will be instrumental to better define which UM biomarkers could be helpful to predict an enhanced positive response to this promising drug. Our increased knowledge on the mechanisms underlying UM development, including aspects of metabolomics [381] and of immune checkpoint inhibition, coupled with novel drug screening efforts taking advantage of robust pre-clinical models will hopefully, in the near future, lead to the development of efficacious therapeutic approaches against UM.

## 9. Conclusions

UM is a rare and complex neoplastic disease with a distinctive biological behaviour. For the majority of patients, the prognosis is dismal after metastases develop in the liver or other sites, since there are currently limited therapeutic strategies for the widespread disease. Indeed, despite extensive research over the past decades our ability to extend the survival of UM patients has not dramatically changed. The continuous efforts to find better estimators of prognosis for UM has led to the recent discovery of novel promising proteins, genomic and proteomic signatures with prognostic implication for UM patients, which complement the list of already well-established prognostic factors in UM. However, a unifying and optimized prognostic model, encompassing clinical and molecular information, widely accepted by the UM community is still lacking. The development of such model is an imminent challenge and could decisively positively impact in a personalized medicine approach, leading to patient-directed surveillance plans and patient-tailored therapies. In this regard, diagnostic methods that allow an earlier disease relapse detection, such as LB, coupled with better pre-clinical models of research which allow a more comprehensive understanding of the UM unique biology, UM metastization and enhanced capacity for in vitro drug testing, as well as a more comprehensive insight on the interplay between different immune infiltrating cells and UM cells will soon help to accelerate the process of therapy development for UM. Hopefully, in the near future we will be able to slow or halt disease progression or even cure UM.

## Figures and Tables

**Table 1:** Most frequent anatomic sites afflicted by metastases in uveal melanoma. The estimation of organ involvement was based on the compilation of the data from 1092 patients extracted from 5 relevant previously published studies [24,41,42,43,44,45]. Relative percentages are variable depending on the cohort studied. However, in all studies, the liver is the preferential site of UM metastization.

Anatomical Site	% of Cases
Liver	85
Lung	29
Bones	16
Subcutaneous tissue	12
Lymph Nodes	11
Brain	5
Other sites	13
Multiple sites	32

**Table 3:** Novel promising prognostic gene signatures in primary uveal melanoma. The novel gene signatures for uveal melanoma described below were published in the past three years.

Study	Gene Signature	Relevance of the Study
Wan et al., 2018 [148]	*ABTB1, ADPRHL1, NTRK2* and *SLC17A7* are hub genes in UM	Important diagnostic and prognostic markers for UM recurrence detection
Xue et al., 2019 [149]	*AC010442.3, AC023790.2, AC092821.1, AL137784.1, CA12, FABP5P1, FAM189A2, GRIN2A, MGLL, MIR4655, MMP9, PARP8, RNF208, S100A13, SIRT3, TCTN1, ZBED1* and *ZNF497*	Early identification of UM patients with poor and good prognosis
Ni et al., 2019 [150]	*ABHD3, APOM, CALHM2, CENPV, CHAC1, HTR2B, HTRA3, LZTFL1, UBE2W, VCPIP1, ZNF391, ZNF415, ZNF667-AS1* and *ZNF835*	Gene signature that allowed prediction of overall survival (OS) and recurrence-free survival (RFS)
Choi et al., 2020 [151]	*CTNNB1, CYC1, NDUFB9* and *NDUFV2* are hub genes in UM	Lower expression of CTNNB1 and increased expression of NDUFB9, NDUFV2 and CYC1 are associated with decreased survival of UM patients
Luo et al., 2020 [152]	*ANXA2P2, CA12, HMCES, POMGNT2, RNF208, SIRT3, SLC44A3, STPG1, TCTN1* and *ULBP1*	High expression of ANXA2P2, CA12 and ULBP1 and a low expression of HMCES, POMGNT2, RNF208, SIRT3, SLC44A3, STPG1 and TCTN1 are associated with higher metastatic risk and a shorter survival
Wan et al., 2020 [153]	*CREG1, HIST1H4E, LZTS1, NIPA1, SH2D3A* and *TMEM201*	Low expression of LZTS1 and TMEM201 plus high expression of CREG1, HIST1H4E, NIPA1, SH2D3A are associated with decreased survival of UM patients
Tang and Cai, 2021 [154]	*EIF1B, MEGF10, PHLDA1, RPL10A, RPL15, SLC25A38, TFDP2 and TIPARP*	Robust prediction model of metastases-free survival
Jun Liu et al., 2021 [155]	*ARPC1B, BTBD6, GUSB, KRTCAP2, RHBDD3* and *SLC39A4*	Robust prediction model of OS for UM patients

**Table 4:** Novel promising prognostic immunohistochemistry-based biomarkers in primary uveal melanoma. The new prognostic markers for uveal melanoma highlighted below were published in the past three years.

Protein	Function	Relevant Conclusions of the Study
**ABCB5** (ATP-binding cassette sub-family B member 5) [184]	P-glycoprotein involved in the transport of molecules across membranes Cancer stem cell marker	Higher expression of ABCB5 is associated with metastases development and worse prognosis
**Adiponectin** [185]	Anti-carcinogenic and insulin-sensitizing actions	Expression of Adiponectin and its receptor Adipor1 was decreased in cases of UM with M3, suggesting that the lower levels of adiponectin could boost the metastatic potential of UM
**ATR** (ataxia telangiectasia and Rad3-related) [186]	Member of the DNA damage response (DDR) protein machinery	Loss of nuclear ATR is associated with well-established markers of poor prognosis in UM (epithelioid cell morphology, increased tumour thickness, higher number of mitotic figures and BAP1 loss)
**ATM** (ataxia-telangiectasia mutated) [187]	Member of the DNA damage response (DDR) protein machinery	Loss of nuclear ATM is associated with well-established markers of poor prognosis in UM (epithelioid cell morphology, large tumour diameter above 10 mm, TILs and nuclear BAP1 loss) and a significant shorter DFS
**Beclin-1** [188]	Protein involved in autophagy	Higher expression of Beclin-1 was correlated with a decreased risk of metastases and increased DFS times
**BNIP3** (BCL2 19 kD protein-interacting protein 3) [189]	Mitochondrial protein involved in regulation of cell death, autophagy and cellular protection	Higher expression of BNIP3 was correlated with a shorter survival
**BTNL9** (Butyrophilin-like protein 9) [190]	Modulator of T-cell mediated immune function	Higher expression of BTNL9 was significantly correlated with a better OS
**c-Rel** [191]	Member of the NF-κB pathway, which regulates a large array of genes implicated in cell survival, inflammatory disorders, response to infection, autoimmune disorders and cancer, among other processes	Nuclear expression of c-Rel expression was significantly associated with inferior survival
**EphA1** (Eph-A1 receptor, erythropoietin-producing human hepatocellular receptor A1) [192]	Member of the Ephrin receptors, which are receptor tyrosine kinases (RTKs) that play a myriad of roles during the embryonic development (for example, in axon guidance, cell migration, segmentation and formation of tissue boundaries) and adulthood (for example, in angiogenesis, stem cell differentiation, regulation of the immune system and in cancer development)	Lower expression of EphA1 is associated with a worse prognosis
**EphA5** (Eph-A5 receptor, erythropoietin-producing human hepatocellular receptor A5) [192]	Member of the Ephrin receptors, which are receptor tyrosine kinases (RTKs) that play a myriad of roles during the embryonic development (for example, in axon guidance, cell migration, segmentation and formation of tissue boundaries) and adulthood (for example, in angiogenesis, stem cell differentiation, regulation of the immune system and in cancer development)	Lower expression of EphA5 is associated with a worse prognosis
**HDAC-2** (Histone Deacetylase 2) [193]	Regulation cellular proliferation, differentiation, angiogenesis and cell death, being implicated in neurodegeneration and cancer	Higher expression of HDAC-2 is an independent factor of better survival in UM
**Nestin** [194]	Intermediate filament protein marker of stem cells in the central nervous system and a cancer stem cell marker	Correlation between nestin positivity and well-established markers of bad prognosis (epithelioid cell morphology, higher mitotic counts, M3 and chromosome 8q gain) Nestin positivity in UM is associated with a worse prognosis
**p50** [195]	Member of the NF-κB pathway, which regulates a large array of genes implicated in cell survival, inflammatory disorders, response to infection, autoimmune disorders and cancer, among other processes	Nuclear immunoreactivity of p50 significantly correlated with metastases development
**p52** [196]	Member of the NF-κB pathway, which regulates a large array of genes implicated in cell survival, inflammatory disorders, response to infection, autoimmune disorders and cancer, among other processes	Expression of p52 was associated with BAP1 loss Higher p52 expression was associated with worse MFS and OS
**p65** [195]	Member of the NF-κB pathway, which regulates a large array of genes implicated in cell survival, inflammatory disorders, response to infection, autoimmune disorders and cancer, among other processes	Nuclear immunoreactivity of p65 significantly correlated with metastases development
**PARP1** [Poly(ADP-ribose) polymerase 1)] [197]	Involved in DNA repair and programmed cell death	Higher expression of PARP-1 is associated with decreased DFS and OS
**PD-1** (Programmed cell death receptor-1) [198]	Involved in immune regulation	High expression of PD-1 in UM cells is associated with decreased DFS and OS
**PD-L1** (Programmed cell death-ligand 1) [199]	Involved in immune regulation	PD-L1 immunoexpression was a significant prognostic factor of a reduced DFS
**PLK-1** (Polio-like kinase 1) [200]	Kinase involved in the regulation of cell cycle	Low expression of PLK-1 was correlated with a higher TNM staging and a significantly decreased OS
**PRDX3** (thioredoxin-dependent peroxidase reductase) [201]	Mitochondria protein with a fundamental role in the antioxidant defence of the cell	High PRDX3 expression is correlated with metastatic disease development and reduced OS
**RelB** [196]	Member of the NF-κB pathway, which regulates a large array of genes implicated in cell survival, inflammatory disorders, response to infection, autoimmune disorders and cancer, among other processes	Expression of RelB was associated with BAP1 loss and with inferior MFS
**SPANX-C** (Sperm protein associated with the nucleus on the X chromosome protein C) [202]	Belongs to a family of proteins expressed in the testis during spermatogenesis	Higher expression of SPANX-C in primary UM is associated with a decreased MFS
